# Neutrophil Gelatinase-Associated Lipocalin: A Shared Early Biomarker of Remote Organ Dysfunction in Blast-Induced Extremity Trauma

**DOI:** 10.3390/ijms26167794

**Published:** 2025-08-12

**Authors:** Cassie J. Rowe, Uloma Nwaolu, Philip J. Spreadborough, Thomas A. Davis

**Affiliations:** 1 Department of Surgery, Uniformed Services University of the Health Sciences, Bethesda, MD 20814, USA; cassie.rowe.ctr@usuhs.edu (C.J.R.); uloma.k.nwaolu@gmail.com (U.N.); 2 The Henry M. Jackson Foundation for the Advancement of Military Medicine, Inc., Bethesda, MD 20817, USA; 3 Academic Department of Military Surgery and Trauma, Royal Centre for Defence Medicine, Birmingham B15 2WB, UK; philip.spreadborough989@mod.gov.uk; 4 Department of Surgery, Torbay and South Devon NHS Foundation Trust, Torquay TQ2 7AA, UK

**Keywords:** polytrauma, inflammation, tourniquet, ischemia/reperfusion injury, remote organ injury

## Abstract

Polytrauma is a critical global health concern characterized by immune dysregulation and a high risk of multiple organ dysfunction syndrome (MODS). Early molecular mechanisms linking trauma severity to organ injury are poorly understood. We used two rat blast-polytrauma models: a tourniquet-induced ischemia/reperfusion injury (tIRI) model and a non-ischemia/reperfusion injury (non-IRI) model. Naïve animals served as controls. RT-qPCR of 120 inflammatory genes in the lung, kidney, and liver, combined with STRING protein–protein interaction analysis, revealed distinct yet overlapping inflammatory gene signatures across all the organs. A core set of genes (*Il6*, *Lbp*, *Nos2*, and *Lcn2*) was consistently upregulated, indicating shared inflammatory pathways. Transcriptomic responses were most pronounced in the tIRI group, with greater magnitude and altered temporal dynamics, uniquely amplifying pro-inflammatory cytokines, immune cell activators, chemokines, and tissue damage markers. Lipocalin-2 (*Lcn2*/NGAL) emerged as a shared hub gene across all the organs within 24 h post-injury. Its expression significantly correlated with MODS activity and adverse outcomes, independent of the injury model. At 168 h, *Lcn2* expression correlated with increased liver damage and NGAL levels correlated with tissue trauma severity. These findings elucidate distinct pro-inflammatory mediators and networks underlying secondary organ dysfunction, highlighting NGAL as a potential universal biomarker of trauma-induced inflammation and MODS activity, suggesting it as a therapeutic target.

## 1. Introduction

Severe traumatic injury remains a leading cause of morbidity, disability, and mortality globally [1,2]. While immediate trauma-related deaths often result from acute blood loss (hemorrhagic shock) and traumatic brain injury, serious secondary complications arise from exaggerated and/or aberrant local and systemic induced inflammatory responses (SIRS). These responses, triggered by hypoxia, organ and soft-tissue injury releasing endogenous damage-associated molecular patterns (DAMPs), infection, and ischemia/reperfusion injury (IRI), can result in secondary multi-organ dysfunction/failure syndromes (MODS or MOFS) [2,3,4,5]. Among trauma patients in the ICU, the development of remote secondary organ damage complications driven by SIRS is proportional to injury severity and accounts for over 50% of delayed mortality and long-term morbidity [6,7]. Challenges persist in the early diagnosis and treatment of organ dysfunction following trauma, largely due to an incomplete understanding of the evolving innate and adaptive immune responses at molecular, cellular, tissue, and organ levels. Addressing this knowledge gap is essential for developing streamlined diagnostics and effective treatments, both in civilian and military practice, where at-risk patients being identified earlier enables appropriate resource allocation, escalation of care, or patient evacuation. Similarly, modulation of these aberrant processes offers potential future therapeutic targets to prevent a dysregulated immune response.

As a common form of severe trauma, polytrauma is particularly associated with complex local and systemic inflammatory cascades that are key determinants of clinical outcomes [2,4,8,9,10,11]. These systemic responses are often amplified by additional insults such as acute IRI, which exacerbate local tissue damage and trigger pathological inflammatory processes characterized by oxidative stress, release of DAMPs, pro-inflammatory mediator release, neutrophil activation, and endothelial/epithelial dysfunction [2,4,12,13,14,15,16,17]. Although data remains limited on the molecular evolution of remote MODS after polytrauma, animal models offer valuable insight into these mechanisms. In our previous work, using a rat model of blast-related traumatic extremity injury, we demonstrated increased risk of remote organ dysfunction (lung, kidney, and liver) following tourniquet-mediated IRI (tIRI) injury and delayed limb amputation [18].

**Figure 1:**
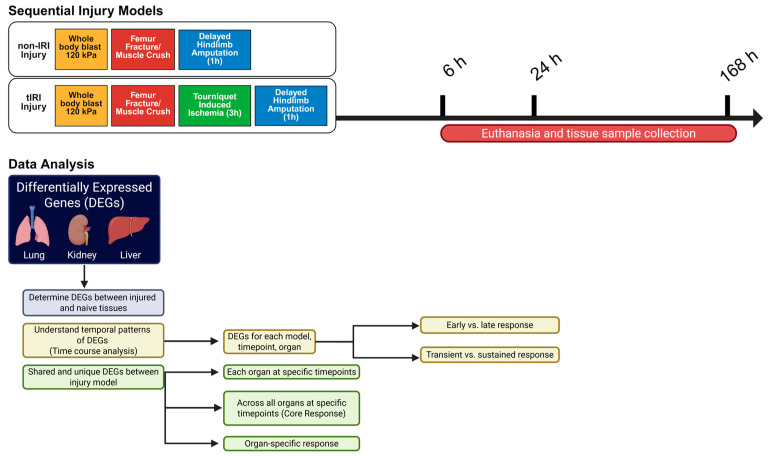
Experimental Strategy and Data Analysis Workflow. Schematic illustration of the research study approach, employing two polytraumatic injury models: a non-ischemia/reperfusion injury (non-IRI) model (whole-body blast, femur fracture/muscle crush, and delayed hindlimb amputation) and a tourniquet-induced ischemia/reperfusion injury (tIRI) model (incorporating tourniquet-induced ischemia before amputation). Tissue samples from the lung, kidney, and liver were collected at 6, 24, and 168 h post-injury. Differentially expressed gene (DEG) analysis was conducted to compare injury groups at each time point and to examine organ-specific and shared genes across time within and between the models. The analysis specifically aimed to identify upregulated genes shared by both injury conditions at the 6 h or 24 h time points, allowing for the differentiation between organ-specific and commonly shared genes. The schematic was created using the Bio-render web interface.

Building on our prior findings, we posit that superimposed tourniquet-induced ischemia/reperfusion injury (tIRI) significantly exacerbates and uniquely alters the molecular inflammatory response in remote organs following blast-induced extremity trauma, contributing to distinct patterns of organ dysfunction. To test this, we analyzed primary transcriptomic datasets collected over seven days post-injury from the lung, kidney, and liver in rat models of blast-polytrauma, both with and without tIRI (Figure 1). This analysis aimed to identify shared and unique gene expression signatures in these organs, characterize the temporal dynamics of these changes, and elucidate key molecular mechanisms and potential biomarkers, such as neutrophil gelatinase-associated lipocalin (NGAL), that contribute to remote organ dysfunction, thereby informing the development of targeted diagnostics and therapies.

## 2. Results

### 2.1. Lung Gene Expression Profiles

**Figure 2:**
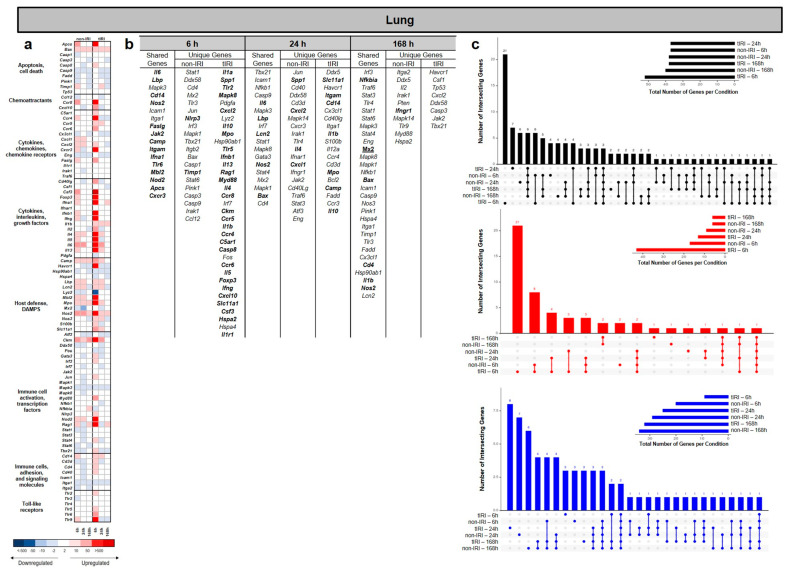
Temporal dynamics of lung gene expression following ischemic and non-ischemic polytrauma. Differentially expressed gene (DEG) analysis in lung tissue at 6, 24, and 168 h post-injury in the non-IRI and tIRI models: (**a**) Heatmap of the selected DEGs, clustered by biological pathway (red = upregulated; blue = downregulated). (**b**) The corresponding list of DEGs is provided, with the upregulated genes relative to naive lung tissue highlighted in bold. (**c**) Vertical UpSet plots summarize differentially expressed genes (DEGs) in the two trauma models across various post-injury time points, detailing the total, upregulated, and downregulated DEGs over time, with the horizontal bars on the inset graph indicating the total regulated genes per group at each time point. The plots also illustrate shared and unique DEGs between the time points, displaying the gene counts above columns and contributing time points listed below.

Superimposed tourniquet-mediated ischemia/reperfusion (tIRI) resulted in significant time-dependent changes in the gene expression profiles in the lung relative to the non-IRI control group (Figure 2). The number of differentially expressed genes (DEGs) changed over the 168 h time course. The UpSet plots in Figure 2c show that the greatest number of significant upregulated and downregulated DEGs were specific to tIRI at the 6 and 24 h time points, respectively. At 6 hpi (Figure 2b), both injury models demonstrated upregulation of *Il6*, *Lbp*, *Cd14*, *Nos2*, *Faslg*, *Jak2*, *Camp*, *Itgam*, *Ifna1*, *Tlr6*, *Mbl2*, *Nod2*, *Apcs*, and *Cxcr3* genes, suggesting an early, robust innate inflammatory response involving pro-inflammatory cytokines, bacterial sensing, leukocyte activation, and cell death pathways. Uniquely, the non-IRI model upregulated *Nlrp3* and *Timp1*, pointing towards inflammasome activation and extracellular matrix modulation. In stark contrast, the tIRI model displayed earlier a significantly more broader and more profound unique upregulation of genes, including numerous pro-inflammatory cytokines (*Il1a*, *Il1b*, *Il5*, *Il13*, *Il4*, *Il10*, *Il1r1*, and *Csf3*), immune cell activators and receptors (*Tlr2*, *Tlr5*, *Myd88*, *Ccr4*, *Ccr5*, *Ccr6*, *Ccr8*, *C5ar1*, and *Slc11a1*), chemokines (*Cxcl2* and *Cxcl10*), interferons (*Ifnb1* and *Ifng*), and factors indicative of tissue damage or repair (*Spp1*, *Mapk8*, *Mpo*, *Ckm*, *Hspa2*, and *Casp8*). Notably, the genes related to lymphocyte development (*Rag1* and *Foxp3*) were also uniquely upregulated. All of this suggests a widespread and more complex dysregulation of both innate and adaptive immune responses. At 24 hpi, the number of shared upregulated genes between the two injury models decreased to five, including *Il6*, *Lbp*, *Lcn2*, *Nos2*, and *Bax*, indicating common pathways involving pro-inflammatory cytokines, bacterial sensing, iron sequestration, nitric oxide production, and apoptosis regulation in both injury contexts. Uniquely, the non-IRI model demonstrated upregulation of *Cxcl2*, *Spp1*, *Il4*, and *Cxcl1*, suggesting a slower/delayed response in neutrophil recruitment, broader inflammatory modulation, and a shift towards Th2-type immune responses. In contrast, the tIRI model uniquely exhibited upregulation of *Il1b*, *Slc11a1*, *Itgam*, *Cd14*, *Mpo*, *Camp*, and *Il10*, suggesting a pronounced pro-inflammatory response, activation of the inflammasome, enhanced bacterial recognition, leukocyte activation and migration, neutrophil-mediated damage, antimicrobial defense, and compensatory anti-inflammatory mechanisms. At 168 hpi, *Mx2*, *Nfkbia*, *Bax*, *Cd4*, *Il1b*, and *Nos2* were the commonly upregulated genes in both injury models. These findings suggest a common set of core genes involved in maintaining sustained responses involving inflammation, apoptosis, T-cell-related processes, and nitric oxide production. The non-IRI model uniquely upregulated *Ifngr1*, indicating a specific ongoing interferon-gamma signaling pathway. Notably, no unique genes were significantly upregulated in the tIRI model at the 168 h time point (Figure 2b).

### 2.2. Kidney Gene Expression Profiles

**Figure 3:**
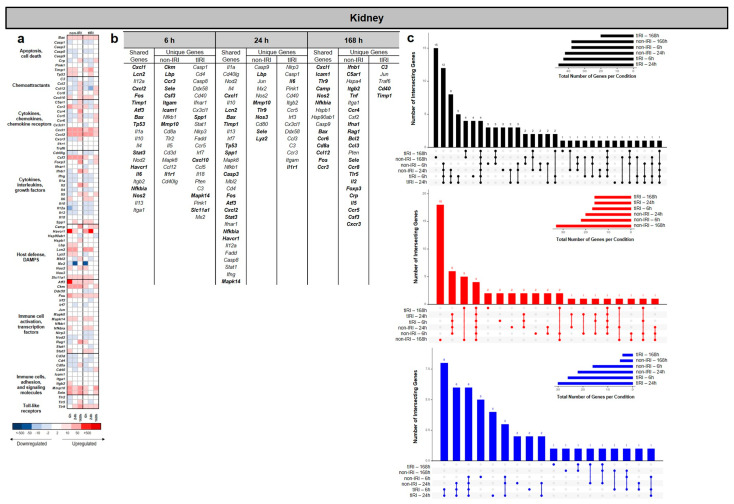
Kidney transcriptomic response to ischemic and non-ischemic polytrauma over time. Differentially expressed gene (DEG) analysis in kidney tissue at 6, 24, and 168 h post-injury in the non-IRI and tIRI models: (**a**) Heatmap of the selected DEGs, clustered by biological pathway (red = upregulated; blue = downregulated). (**b**) The corresponding list of DEGs is provided, with the upregulated genes relative to naive kidney tissue highlighted in bold. (**c**) Vertical UpSet plots summarize differentially expressed genes (DEGs) in the two trauma models across various post-injury time points, detailing the total, upregulated, and downregulated DEGs over time, with the horizontal bars on the inset graph indicating the total regulated genes per group at each time point. The plots also illustrate shared and unique DEGs between the time points, displaying the gene counts above columns and contributing time points listed below.

Consistent with the lung data, the gene expression analysis revealed that tIRI induced a significant and time-dependent alteration in the kidney transcriptome compared to the non-IRI control (Figure 3). The number of differentially expressed genes (DEGs) changed over the 168 h time course. The UpSet plots in Figure 3c show that the greatest number of significant regulated DEGs were specific to tIRI at 6 and 24 hpi. Across all the time points, the tIRI group at these time points exhibited a greater number of downregulated genes, whereas the greatest number of significantly upregulated genes was measured in the non-IRI group at 168 hpi (Figure 3b,c). While a subset of DEGS was shared between the two injury conditions, a substantial number were uniquely expressed in each group, alongside a set of DEGs shared between the two groups (Figure 3b). At 6, 24, and 168 hpi, we analyzed the kidney samples from the non-IRI and tIRI models using the heatmap and upregulated DEGs (Figure 3a,c) to identify shared and unique gene expression. At 6 hpi both injury models exhibited upregulation of several shared genes: *Cxcl1*, *Lcn2* (a known kidney injury marker), *Cxcl2*, *Fos*, *Timp1* (biomarker of kidney injury), *Atf3*, *Bax*, *Tp53*, *Stat3*, *Havcr*/(*Kim1*) (another established kidney injury biomarker), *Il6*, *Nfkbia*, and *Nos2.* Collectively, expression of these genes indicates an early and robust common injury response involving chemokine-mediated recruitment, innate immunity, cell stress, apoptosis, and inflammation. The non-IRI model uniquely upregulated *Ckm*, *Lbp*, *Ccr3*, *Sele*, *Csf3*, *Itgam*, *Icam1*, *Mmp10*, and *Il1r1*, pointing towards sustained leukocyte adhesion, recruitment, and extracellular matrix remodeling. In contrast, the tIRI model uniquely upregulated *Spp1*, *Cxcl10*, *Mapk14*, and *Slc11a1*, suggesting a more focused acute inflammatory response driven by *Spp1*, specific chemokine signaling, and stress pathway activation. By 24 hpi, shared upregulated genes included *Cxcl1*, *Lcn2*, *Bax*, *Timp1*, *Tp53*, *Spp1*, *Casp3*, *Fos*, *Atf3*, *Cxcl2*, *Stat3*, *Nfkbia*, *Havcr1*, and *Mapk14*. Expression of these genes suggests active chemokine-mediated recruitment, apoptosis, tissue remodeling, stress responses, and inflammatory signaling. Uniquely, the non-IRI model continued to show upregulation of *Lbp*, *Mmp10*, *Tlr9*, *Nos3*, *Sele*, and *Lyz2*, suggesting sustained bacterial recognition, extracellular matrix degradation, endothelial activation, and innate immune responses. In contrast, the tIRI model uniquely upregulated *Il6* and *Il1r1*, pointing towards a specific exacerbation of central pro-inflammatory cytokine pathways in response to the ischemia/reperfusion insult. At 168 hpi shared genes included *Cxcl1*, *Icam1*, *Tlr9*, *Camp*, *Nos2*, *Nfkbia*, *Bax*, *Ccr6*, *Cd8a*, *Ccl12*, *Fos*, and *Ccr3.* This sustained expression implies persistent involvement in chemokine signaling, leukocyte adhesion, bacterial recognition, antimicrobial defense, inflammation regulation, apoptosis, and T-cell-mediated processes. Uniquely, the non-IRI model demonstrated a broad and complex upregulation of genes, including interferons (*Ifnb1* and *Ifna1*), complement pathway components (*C5ar1*), integrins (*Itgb2*), pro-inflammatory cytokines (*Tnf*, *Il2*, and *Il5*), various chemokine receptors (*Ccr4*, *Ccr8*, *Tlr5*, *Ccr5*, and *Cxcr3*), lymphocyte development and regulation markers (*Rag1*, *Bcl2*, and *Foxp3*), chemokines (*Ccl3*), acute-phase markers (*Crp*), and leukocyte activators (*Sele* and *Csf3*). This pattern is suggestive of a widespread and adaptive immune response. In contrast, the tIRI model uniquely upregulated *C3* (complement component 3), *Cd40*, and *Timp1*, pointing towards a more specific and sustained activation of the complement system, immune cell co-stimulation, and renal tissue and remodeling processes.

### 2.3. Liver Gene Expression Profiles

**Figure 4:**
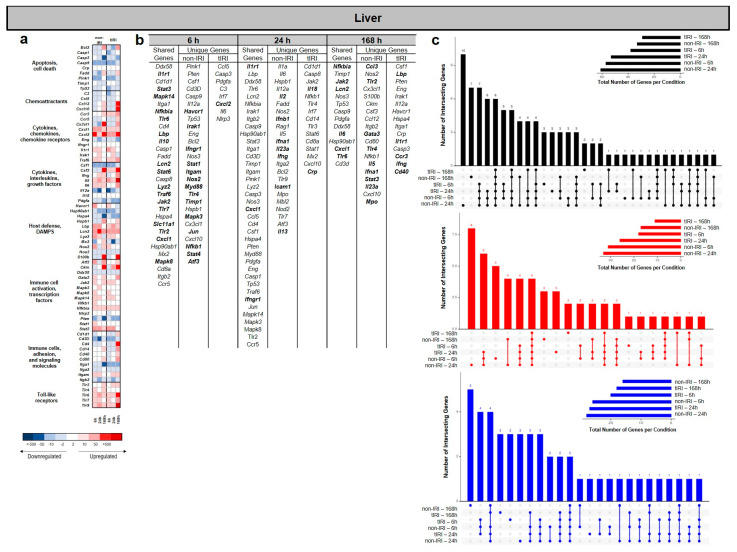
Liver transcriptomic response to ischemic and non-ischemic polytrauma over time. Differentially expressed gene (DEG) analysis in liver tissue at 6, 24, and 168 h post-injury in the non-IRI and tIRI models: (**a**) Heatmap of the selected DEGs, clustered by biological pathway (red = upregulated; blue = downregulated). (**b**) The corresponding list of DEGs is provided, with upregulated genes relative to naive liver tissue highlighted in bold. (**c**) Vertical UpSet plots summarize differentially expressed genes (DEGs) in the two trauma models across various post-injury time points, detailing the total, upregulated, and downregulated DEGs over time, with the horizontal bars on the inset graph indicating the total regulated genes per group at each time point. The plots also illustrate shared and unique DEGs between the time points, displaying the gene counts above columns and contributing time points listed below.

In contrast to the findings observed in the lung and kidney, the liver tissue from the nontIRI group showed the greatest number of significantly upregulated and downregulated genes at 24 hpi regulated genes across all the time points (Figure 4a–c), except where the number of upregulated genes in the tIRI group was modestly higher at 168 hpi (Figure 4c). While some DEGs were shared between models, many were uniquely regulated in each condition (Figure 4b,c). The heatmap and gene list analysis (Figure 4a,b) revealed that at 6 hpi, both injury models shared upregulation of genes involved in general inflammatory and immune responses, cell signaling (JAK/STAT and MAPK pathways), and innate immunity. These shared genes include *Il1r1*, *Stat3*, *Mapk14*, *Lcn2*, *Stat6*, *Lyz2*, *Traf6*, *Jak2*, *Tlr7*, *Slc11a1*, *Tlr2*, *Cxcl1*, and *Mapk8*. The non-IRI model uniquely upregulated a broader set of genes, including *Havcr1*, *Irak1*, *Ifngr1*, *Stat1*, *Itgam*, *Nos2*, *Myd88*, *Tlr4*, *Timp1*, *Mapk3*, *Jun*, *Nfkb1*, *Stat4*, and *Atf3*. This indicates a more diverse activation of immune pathways, encompassing additional pattern recognition receptors, interferon responses, and stress-inducible factors. In contrast, the tIRI model uniquely upregulated only *Cxcl2*, a potent neutrophil chemoattractant. This highly specific response suggests a more pronounced or targeted neutrophil recruitment driven by the ischemia/reperfusion component of the injury [19,20]. By 24 hpi, liver tissue gene expression revealed sustained shared upregulation of *Il1r1*, *Cxcl1*, and *Ifngr1* in both models, indicating common inflammatory and chemokine signaling. Distinctly, the non-IRI model uniquely upregulated several genes involved in interferon and T-helper cell-mediated immunity (*Il2*, *Ifnb1*, *Ifna1*, *Il23a*, *Ifng*, and *Il13*), alongside cell adhesion (Icam1), suggesting a broad, ongoing adaptive immune response. In contrast, the tIRI model uniquely upregulated *Il18* and *Crp*, highlighting a specific and potentially more severe pro-inflammatory and acute-phase response characteristic of significant tissue damage. At 168 hpi, both models showed distinct gene expression profiles. Shared upregulated genes, including *Nfkbia*, *Jak2*, *Lcn2*, *Il6*, *Cxcl1*, and *Tlr6*, indicated sustained common involvement in inflammation regulation, cytokine signaling, and innate immunity. The non-IRI model uniquely upregulated *Ccl3*, *Tlr2*, *Gata3*, *Tlr4*, *Il5*, *Ifna1*, *Stat3*, *Il23a*, and *Mpo*, pointing towards a persistent Th2- and innate immunity-driven response. Conversely, the tIRI model uniquely exhibited upregulation of *Il1r1*, *Lbp*, *Ccr3*, *Ifng*, and *Cd40*, suggesting a distinct, prolonged inflammatory and immune cell activation, potentially involving bacterial recognition and T-cell mediated processes.

**Figure 5:**
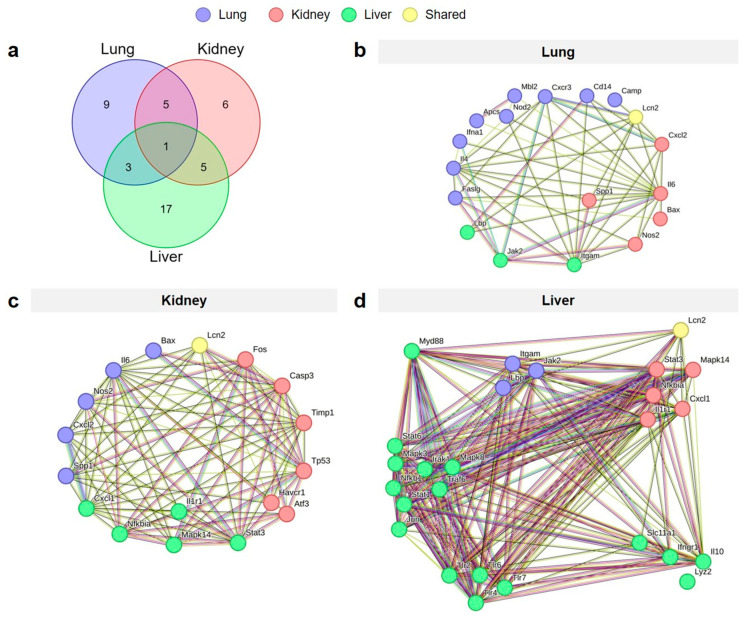
Shared and organ-specific upregulated genes across lung, kidney, and liver tissues following polytrauma injury. The Venn diagram (**a**) illustrates the distribution of genes unique to each organ and those shared across organs. Gene network visualizations of the upregulated unique genes specific to the lung (**b**), kidney (**c**), and liver (**d**), and those shared between organs. Nodes within each network represent the upregulated genes, and edges depict interactions between them.

Analysis of the upregulated genes across the lung, kidney, and liver tissues in both the non-IRI and tIRI groups revealed a complex interplay of shared inflammatory responses (Figure 5). A Venn diagram illustrates the number of upregulated genes unique to each organ and those shared between them at 6–24 hpi. Specifically, 9 genes were uniquely upregulated in the lung, 6 in the kidney, and 17 in the liver. Notably, one gene, *Lcn2*, was consistently upregulated across all three organs in both injury groups. Five genes (*Cd14*, *Camp*, *Aoc1*, *Faslg*, *Bax*) were shared between the lung and kidney. The lung and liver shared three genes: *Il1b*, *Cxcl10*, and *Ptgs2*. The kidney and liver shared five genes (*Saa3*, *C3*, *Lbp*, *Hp*, and *Irf7*). String gene network analysis of the shared upregulated genes in each organ highlights the potential associated functional relationships between genes and predicted protein–protein interaction.

### 2.4. Systemic and Cross-Organ NGAL Responses

**Figure 6:**
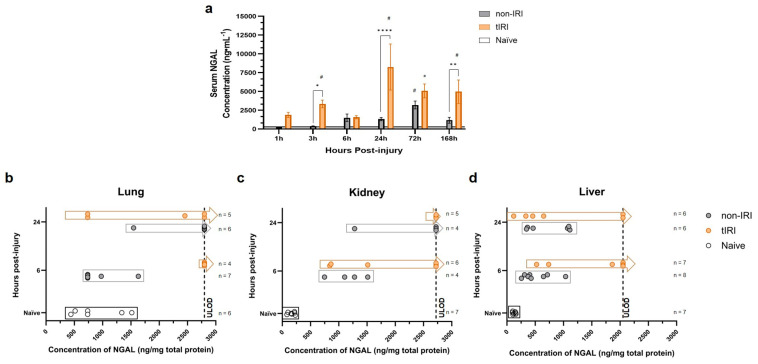
Neutrophil gelatinase-associated lipocalin (NGAL) levels following polytrauma with or without tourniquet-induced ischemia/reperfusion injury. NGAL concentrations were quantified in the samples collected following either non-ischemia/reperfusion injury (non-IRI) or tourniquet-induced ischemia/reperfusion injury (tIRI) polytrauma models using a commercial ELISA kit (ab119602, Abcam): (**a**) Serum NGAL levels measured at 1, 3, 6, 24, 72, and 168 h post-injury. Serum samples were diluted between 1:800 and 1:4000 based on preliminary testing, and final concentrations were calculated accounting for dilution factors. (**b**–**d**) NGAL levels in tissue lysates prepared from organ samples collected at 6 and 24 h post-injury (corresponding to time points for DEG analysis). Approximately 100 mg of each tissue sample was homogenized in RIPA buffer containing protease and phosphatase inhibitors. Tissue NGAL concentrations were normalized to total protein concentration, which was determined using a BCA protein assay. Optical density was read at 450 nm, and concentrations were interpolated from a five-parameter logistic (5-PL) standard curve. All the assays were performed in duplicate, and average values were used for analysis. The hashed vertical lines in panels (**b**–**d**) mark the upper limit of detection (ULOD) for the assay. The samples with concentrations exceeding this limit, for which precise values could not be determined, are plotted to the right of the hashed line. Asterisks (*) represent significant changes in NGAL concentration between injury models in varying level of significance where, * = *p* < 0.05, ** = *p* < 0.01, and **** = *p* < 0.0001. Hashtags (#) represent significant changes in NGAL concentrations from naïve, uninjured animals.

As a next step in our investigation, circulating NGAL levels were measured at 1, 3, 6, 24, 72, and 168 hpi (Figure 6a). The tIRI cohort exhibited a significant and progressive increase in NGAL levels over the first 24 h, and remained significantly elevated at 168 hpi, highlighting the prolonged and sustained inflammatory mediator/LCN response from the tIRI over the nontIRI group. In contrast, the non-IRI cohort showed a delayed and attenuated NGAL elevation. Despite these differences, the NGAL levels remained significantly elevated in both cohorts at 168 h, with the tIRI group demonstrating higher concentrations.

To validate gene–protein correlation, the tissue NGAL protein levels were quantified in lysates from the lung, kidney, and liver at 6 and 24 hpi (Figure 6b–d). NGAL protein production in all three tissues increased significantly following trauma. This increase correlated with trauma severity and mirrored the observed gene expression patterns.

## 3. Discussion

This study analyzed primary transcriptomic datasets from the lung, kidney, and liver over a seven-day period following blast-related traumatic extremity injury in a rat model [18]. The analysis compared outcomes with and without superimposed tourniquet-mediated ischemia/reperfusion (tIRI) to identify shared and unique time-varying gene expression signatures in these organs post-trauma, thus providing insights into the molecular mechanisms of trauma-induced remote end-organ dysfunction.

Our findings demonstrate that severe blast-related extremity trauma, especially when compounded by tIRI, elicits a complex and dynamic inflammatory response across multiple organs, including the lung, kidney, and liver. A core set of genes, such as *Il6*, *Lbp*, *Nos2*, *Fos*, *Atf3*, *Stat3*, *Jak2*, *Nfkbia*, *Bax*, *Tp53*, *Camp*, and *Lcn2*, are consistently upregulated in both non-IRI and tIRI models, signifying shared pathways of inflammation, bacterial sensing, nitric oxide production, and stress-induced cell death. tIRI uniquely amplifies and broadens this inflammatory signature. However, tIRI leads to a more profound and widespread upregulation of pro-inflammatory cytokines like *Il1b*, *Il1a*, *Il5*, *Il13*, *Il4*, and *Il10*; immune cell activators and receptors such as *Tlr2*, *Tlr5*, *Myd88*, *Ccr4*, *Ccr5*, *Ccr6*, *Ccr8*, *C5ar1*, and *Cd40*; and chemokines including *Cxcl2* and *Cxcl10*. Furthermore, tIRI uniquely elevates interferons like *Ifnb1* and *Ifng*, and markers of severe tissue damage and neutrophil activity such as *Mpo* and *Spp1*. This distinct gene expression profile underscores the significant impact of tourniquet-induced ischemia/reperfusion in exacerbating systemic inflammation and tissue damage beyond that caused by trauma alone, with organ-specific responses evolving over time. We speculate the tIRI amplified response likely stems from an intensified inflammatory cascade initiated by local DAMP release from the site of extremity injury [14,21,22] and subsequent systemic mediators (cytokines and chemokines) [9,23,24,25]. Excessive local production of these factors, particularly in the tIRI group, may exceed local containment, leading to systemic spillover [10,11,23]. These circulating mediators can then activate immune and endothelial cells in distant organs, propagating inflammation [8,26,27]. Additionally, circulating DAMPs (HMGB1 and mtDNA) can directly stimulate systemic inflammation [26,27], which can induce cell stress and death in remote organs, potentially causing further DAMP release. This process can create a positive feedback loop that sustains and escalates systemic inflammation [12,16,17,23], representing an exaggerated host response to the combined injury [10]. In severe cases, this dysregulation can manifest as a hyperinflammatory state or cytokine storm [25,28] driven by DAMP-initiated feedback. This intensified systemic mediator activity, amplified by spillover and feedback, likely underlies the greater molecular perturbation observed in end organs [11,12,14,18,29,30,31], potentially contributing to subsequent inflammation, damage, and dysfunction.

Consistent with the findings from other polytrauma studies, our results underscore the importance of specific and evolving inflammatory gene signatures in end organs following severe trauma. These signatures reflect distinct molecular signaling events that drive organ-specific pathological outcomes. We propose that these signatures develop because each organ processes systemic inflammatory mediators such as DAMPs and cytokines differently, based on its unique cellular makeup, microenvironment, and regulatory control of pathways like NF-κB, MAPK, and JAK-STAT. Therefore, temporal shifts in organ-specific gene expression may determine the extent of local inflammation, tissue damage, fibrosis, and functional decline, contributing to the clinical variability seen in MODS. Our data support a view of MODS as a complex outcome of varied, interacting organ-level pathologies, rather than a uniform systemic failure. This perspective suggests opportunities for developing more precise molecular signature-based diagnostics and prognostics, and for targeted therapies that modulate specific detrimental pathways within individual organs, potentially improving efficacy and reducing side effects compared to broad systemic treatments.

Building on this view of distinct organ-level responses to systemic inflammation and the potential for targeted therapies, our findings specifically highlight the contribution of neutrophil gelatinase-associated lipocalin (NGAL/*Lcn2*). Our findings reinforce the multifaceted role of NGAL as a key mediator in the early inflammatory response to polytrauma, particularly exacerbated by tourniquet-induced ischemia/reperfusion injury, which promotes sterile inflammation. Consistent with its known pro-inflammatory actions contributing to SIRS [32], neuroinflammation [33,34], and traumatic lung injury [32,35], we found a significant correlation between *Lcn*2 and NGAL tissue gene and protein expression across multiple organs (lung, kidney, and liver), circulating protein levels, and overall trauma severity [18]. These findings underscore NGAL not only as a potential biomarker reflecting systemic inflammatory burden and predicting clinical outcomes [36,37,38] but also as a mediator of inter-organ crosstalk, suggested by observed shared and unique gene expression patterns following extremity trauma. Given its predominantly pro-inflammatory role and marked upregulation post ischemia/reperfusion, particularly in the context of acute kidney injury progression [39], NGAL represents a compelling therapeutic target. However, we did observe a transient dip in serum NGAL at 6 h in the tIRI cohort (Figure 6a), despite the overall post-trauma elevation. While the exact reasons for this specific dip are not fully clear from our current data, we speculate it could be due to rapid tissue uptake or clearance of NGAL during an acute compensatory phase, or differing kinetics of NGAL production and release across various injured organs. Further time-course analysis would be beneficial to definitively explain such transient fluctuations. Although it is recognized as a biomarker of renal injury, NGAL appears to play a protective role in the recovery process following ischemia/reperfusion, by potentially promoting cell regeneration, though the precise mechanisms of this protection are still under investigation [39,40,41,42]. Modulating its activity could mitigate detrimental inflammation while preserving beneficial effects. For instance, the natural compound berberine has been shown to alleviate neuroinflammation in part by downregulating the *NFκB/Lcn2* pathway [43], and research into small molecule inhibitors directly targeting NGAL is underway in other disease contexts [44]. Further investigation into NGAL inhibition may offer a valuable approach to improve outcomes following severe trauma and associated systemic inflammation.

Our findings underscore the significant clinical applicability of NGAL as a predictive biomarker for multi-organ dysfunction. In our model, elevated NGAL concentrations consistently precede the onset of classic clinical biomarkers (e.g., BUN, ALT, AST, and Cr), serum inflammatory mediators (e.g., CRP and IL-6), hematological changes, and histological evidence of tissue damage [18,29,45]. This temporal dissociation offers a critical window for early intervention, a crucial advantage given the diagnostic challenges posed by the simultaneous or sequential nature of MODS following trauma. Serum NGAL emerges as an early and more pronounced predictor of injury severity and progression of organ dysfunction, addressing limitations of existing diagnostic tools that often fail to accurately reflect the patient’s clinical course. Furthermore, by elucidating NGAL-mediated pathways, such as those involved in tubular repair and inflammation resolution, our work positions NGAL not only as a vital tool for early identification but also as a legitimate target for novel therapeutic strategies aimed at mitigating multi-organ injury.

These comprehensive insights into the complex genomic landscape post-trauma offer a robust foundation for developing novel prognostic clinical biomarkers for critical complications like MODS and pave the way for precision medicine strategies. Our study delved into the intricate interplay of shared and unique gene expression signatures across various end organs following traumatic injury, aiming to elucidate both systemic and organ-specific molecular responses. We found distinct temporal patterns of gene activation, showing that the body’s reaction changes significantly from the hyperacute phase to later stages post-injury. Specifically, we identified conserved inflammatory and repair pathways, such as those related to immune cell activation, stress response, and tissue regeneration, which are universally activated across affected organs [46,47]. Concurrently, our analysis uncovered unique transcriptional profiles that indicate organ-specific pathology and compensatory mechanisms, reflecting the specialized cellular responses and vulnerabilities of individual tissues [48]. Furthermore, these insights also suggest potential organ crosstalk [30,31,49,50,51,52], a phenomenon where the molecular state and inflammatory mediators released from one injured organ influence the gene expression and overall response of other distant organs following trauma, potentially exacerbating or mitigating overall systemic dysfunction. This detailed understanding of the dynamic and multifaceted genomic landscape post-trauma provides a critical stepping stone for advancing diagnostics and therapeutics in trauma care.

This study has several limitations. First, the sequential induction of polytrauma extremity injuries over a 2.5–3.0 h period does not fully replicate the simultaneous and often unsedated nature of real-world trauma, potentially limiting the direct applicability of our findings. Second, the exclusive use of male Sprague Dawley rats of a specific age restricts the generalizability, as sex and age-related differences in immune responses to trauma are well documented [11,53,54]. Third, a key limitation of this study lies in its focused bioinformatic analysis. We utilized a highly curated panel of 120 inflammation-related genes, a priori selected based on extensive literature review and primer availability. While this approach allowed for a targeted investigation into known mediators of inflammatory responses in trauma, it offered only a narrow view of the broader molecular mechanisms driving secondary organ injury. Furthermore, the analysis was conducted in a relatively small cohort per time point. Future studies employing expanded transcriptomic profiling and increased sample sizes would significantly improve statistical power and the robustness of gene expression analysis, providing a more comprehensive understanding of these complex biological processes. Despite these limitations, our findings provide valuable initial insights into the molecular networks underlying trauma-induced systemic inflammation and remote organ dysfunction, informing the development of potential therapeutic interventions.

In conclusion, this study highlights that severe blast-related extremity trauma, particularly when compounded by tIRI, profoundly exacerbates systemic inflammation and tissue damage in distant organs like the lung, kidney, and liver. Our research reveals that while shared inflammatory pathways are activated by trauma alone, tIRI uniquely amplifies and broadens this inflammatory signature, leading to a more widespread upregulation of pro-inflammatory cytokines, immune cell activators, and markers of severe tissue damage. This intricate interplay of organ-specific and generalized inflammatory responses underscores the complexity of MODS, emphasizing the need for targeted, rather than broad, therapeutic strategies. Notably, we identify the early expression of *Lcn2/Ngal* as a consistently upregulated mediator across affected organs, proposing its potential as both a biomarker for injury severity and a compelling therapeutic target to mitigate detrimental inflammation while preserving beneficial effects. Future research should focus on further elucidating the precise mechanisms by which NGAL contributes to inter-organ crosstalk and exploring specific interventions, such as NGAL modulators, to improve outcomes following severe trauma and systemic inflammation.

## 4. Materials and Methods

### 4.1. Animals

Adult male Sprague Dawley rats (400–550 g, *n* = 49) were obtained from Taconic Farms and acclimated for one week prior to experimentation. All the rats were paired and housed in standard plastic cages with a 12 h light/dark cycle; provided unlimited access to standard rodent chow, fresh water, and enrichment; and acclimated for at least three days prior to experiments, returning to clean cages with soft bedding post-injury. All the procedures were approved by the Uniformed Services University IACUC (protocol #: SUR-20-997 approved on 6 January 2020) and conducted in accordance with the ARRIVE guidelines [18,55] and all the applicable Federal regulations governing the protection of animals in research. To identify significant changes in gene expression and manage outliers, a sample size of *n* = 7 per group was determined. This decision was based on historical data from our trauma models, providing 80% statistical power to detect a 25% change in serum clinical markers of injury (e.g., BUN, Cr, AST, and ALT) and inflammatory mediators (e.g., IL-1β, IL-6, and CXCL1) with an alpha of 0.05 [18,29,45].

### 4.2. Polytrauma Models, Animal Procedures, and Tissue Collection

Models: The rats were randomized into three groups. The non-IRI group (*n* = 21) underwent blast overpressure exposure (BOP; 120 ± 7 kPa) in a pneumatically driven shock tube [18,29] and complex extremity injury (CEI; unilateral femur fracture, 1 min soft tissue crush [20 psi]), followed by delayed hindlimb amputation (dHLA) at 60 min post-injury. The tIRI group (*n* = 21) underwent the same BOP+CEI protocol, with the addition of 180 min of tourniquet-induced hindlimb ischemia (250–300 mmHg) immediately proximal to the femur fracture, followed by dHLA [18]. A group of naïve animals (*n* = 7) served as healthy, non-injured controls and did not undergo any procedures.

As previously reported, all the rats in the non-IRI group survived, whereas 33% of the rats in the tIRI group died within the first 72 h, with no deaths observed between 72 to 168 h post injury (hpi) [18].

Anesthesia and Analgesia: The rats were initially anesthetized with 4% isoflurane, followed by an intraperitoneal (IP) injection of ketamine-xylazine (75 mg/kg ketamine (Henry Schein Animal Health, Dublin, OH, USA), 10 mg/kg xylazine (Akorn, Inc., Lake Forest, IL, USA)) for the duration of the procedure, with re-dosing as necessary. For pain management, all the rats received preoperatively sustained-release buprenorphine (1.2 mg/kg; Zoopharm, Windsor, CO, USA) with a repeat dose three days post-injury. The rats were assessed twice daily for three days post-injury using IACUC-approved pain charts.

Blast and Complex Extremity Injury (BOP+CEI): The rats in the non-IRI and tIRI groups were subjected to blast overpressure (BOP) and complex extremity injury (CEI). BOP was administered in an Advanced Blast Simulator overpressure shock tube (ORA Inc., Fredericksburg, VA, USA) at a peak overpressure of 120 ± 7 kPa. Immediately following blast exposure, CEI was induced in the hindlimb. This involved a drop-weight femur fracture using a 581-gram weight dropped from 88 cm via a Lateral Long Bone Ballistic System (University of Alabama-Birmingham, Birmingham, AL, USA), followed by a 1 min soft tissue crush injury at 138 kPa (20 psi), with pressure determined using a Chatillon DF series force gauge (AMETEK Inc., Berwyn, PA, USA).

Tourniquet-Induced Ischemia/Reperfusion Injury: For the tIRI group, a pneumatic tourniquet (Hokanson, Bellevue, WA, USA) inflated to 250–300 mmHg was applied immediately proximal to the femur fracture for 180 min to induce hindlimb ischemia. Limb elevation was performed prior to tourniquet application to reduce venous pooling. Tourniquet inflation and control were managed using a segmental cuff selector and an aneroid sphygmomanometer. This pressure was chosen to ensure complete occlusion and simulate combat application tourniquets.

Delayed Hindlimb Amputation (dHLA): Following BOP+CEI, the rats in the non-IRI group underwent delayed hindlimb amputation (dHLA) through the zone of injury 60 min post-injury. For the tIRI group, dHLA was performed through the zone of injury immediately after 180 min of tourniquet-induced ischemia, followed by 60 min of limb reperfusion. Amputation included appropriate hemostasis and debridement of devitalized tissue and bone fragments, followed by hamstring and quadriceps myoplasty over the exposed residual femur as previously described [18,29]. A separate sterile field and dissection kit were used for each animal during amputation, and closure was completed in a layered fashion.

Tissue Collection: Blood collected in serum separator tubes was obtained by either tail vein venipuncture or cardiac puncture at 1, 3, 6, 24, 72, and 168 hpi. Separated serum was aliquoted and stored at −20 °C. At 6, 24, and 168 hpi, seven rats per group per time point were euthanized, and lung, kidney, and liver tissue biopsies were collected and snap-frozen.

### 4.3. Total RNA Isolation and RT-qPCR

Total RNA was extracted using RNeasy mini kits with on column DNase Digest (Cat#: 217004; Qiagen, Germantown, MD, USA), quantified (NanoDrop, ThermoFisher Scientific, Waltham, MA, USA), and converted to cDNA (Cat #: 1725038; iScript Advanced kits, Bio-Rad, Hercules, CA, USA). We methodologically selected 120 inflammation-related genes for their established roles as key mediators in trauma and multi-organ injury, a decision guided by an extensive literature review. We then prioritized genes with validated qPCR primers to ensure robust and reproducible analysis. This targeted approach allowed for a focused investigation of critical inflammatory pathways within our experimental design [18]. While providing a robust analysis of these specific markers, we acknowledge that this offered a narrow view of the broader transcriptome. The annotations for each gene are listed in Supplemental Table S1. Gene transcript expression (mRNA) was analyzed by RT-qPCR (Cat #: 1725275; SsoAdvanced Universal SYBR Green Supermix, Bio-Rad) on a QuantStudio-7 Pro system. Gene expression was normalized to organ-specific housekeeping genes (lung: *Rplp2*, kidney: *Rplp2*, and liver: *Hprt*) as determined by a comprehensive analysis and expressed as 2^−ΔΔCt^ relative to naïve controls [45].

### 4.4. Protein–Protein Interaction Analysis

Protein–protein interaction (PPI) networks were constructed using STRING (version 12.0; https://string-db.org, accessed on 24 April 2025) to visualize interconnected, significantly upregulated genes in all the organs at 6 and 24 hpi.

### 4.5. NGAL Analysis

Serum and organ lysate levels of neutrophil gelatinase-associated lipocalin (NGAL) were quantified using a commercially available enzyme-linked immunosorbent assay (ELISA) kit (ab119602, Abcam, Boston, MA, USA). For serum analysis, the samples collected at 1, 3, 6, 24, 72, and 168 hpi were thawed on ice and diluted with the kit-provided diluent, with dilutions ranging from 1:800 to 1:4000 based on preliminary experiments [45].

For tissue lysates, the organ samples were weighed (100 ± 10 mg, homogenized in 1 mL of radioimmunoprecipitation assay (RIPA) lysis buffer (ThermoFisher Scientific), and supplemented with 1% (*v*/*v*) protease inhibitor cocktail and phosphatase inhibitor cocktail (Millipore Sigma, Burlington, MA, USA). Total protein concentration was determined using the Pierce bicinchoninic acid (BCA) Protein Assay Kit (ThermoFisher Scientific) by measuring absorbance at 562 nm with an Infinite M Nano microplate spectrophotometer (Tecan, Mannedorf, Switzerland). Sample protein concentrations were calculated by comparing the sample absorbance value to a bovine serum albumin (BSA) standard curve. The NGAL ELISA was performed according to the manufacturer’s instructions, and the optical density (O.D.) was read at 450 nm. A five-parameter logistic (5-PL) curve fitting algorithm was used to generate standard curves, and sample NGAL concentrations were interpolated, with appropriate corrections for dilution factors (for serum) or normalization to total protein (for tissue). All the assays were performed in duplicate, and average values were used for statistical analysis.

### 4.6. Statistical Analysis

Differential gene expression was visualized using heatmaps. Significantly expressed differentially expressed genes (DEGs, >2.0 fold expression) compared to naïve controls (*p* < 0.05) were identified using *t*-tests for each time point and injury pattern. Venn diagrams (Molbiotools, https://molbiotools.com/listcompare.php, accessed on 24 April 2025) were used to visualize shared and unique genes within organs over time, organ-specific unique genes within injury models over time, and shared/unique genes across all the organs and injury conditions. SPSS (version 28.0.1.0) was used for the calculations and statistical analyses. The specific statistical tests used for each comparison are detailed in the Section 2. To identify differentially expressed genes common to the injury models at each time point, we used UpSet plots. These plots were generated using the UpSetR analysis software package (version 1.4.0) in R version 4.4.2 to explore the intersection of genes across the different models and timepoints.

## Data Availability

The datasets used and/or analyzed during the current study are available from the corresponding author upon reasonable request.

## Supplementary Materials

The following supporting information can be downloaded at https://www.mdpi.com/article/10.3390/ijms26167794/s1.

## Abbreviations

The following abbreviations are used in this manuscript:

ARRIVE	Animal research: reporting of in vivo experiments
BCA	Bicinchoninic acid
BOP	Blast overpressure
cDNA	Complementary DNA
DAMPs	Damage-associated molecular patterns
DEGs	Differentially expressed genes
dHLA	delayed hindlimb amputation
ELISA	Enzyme-linked immunosorbent assay
hpi	hours post injury
IACUC	Institutional animal care and use committee
MODS	Multi-organ dysfunction syndrome
MOFS	Multiple organ failure syndrome
mRNA	Messenger RNA
NGAL	Neutrophil gelatinase-associated lipocalin
non-IRI	Injury model minus tourniquet-induced ischemia insult
O.D.	Optical density
PPI	protein–protein interaction networks
qRT-PCR	Quantitative real-time reverse transcription polymerase chain reaction
RIPA buffer	Radioimmunoprecipitation assay buffer
tIRI	Tourniquet-induced ischemia/reperfusion injury model
