# Supplementary material for: Neutrophil Gelatinase-Associated Lipocalin: A Shared Early Biomarker of Remote Organ Dysfunction in Blast-Induced Extremity Trauma
Source: Int J Mol Sci. 2025 Aug 12;26(16):7794. doi: 10.3390/ijms26167794 (PMC12386635; doi:10.3390/ijms26167794)
Supplement: Supplementary file 1 [file ijms-26-07794-s001.zip › ijms-3748128-supplementary.pdf]

Supplemental Table S1. Target Gene List for Innate and Adaptive Immune Response Arrays. This table consists of the target gene name, the gene symbol, RefSeq ID, Bio-Rad assay ID, amplicon length, and the amplicon context sequence provided by Bio-Rad. The gene descriptions were obtained from GeneCards (genecards.org).

Gene Symbol	Gene Name	Gene Description	RefSeq Accession No	Ensembl ID	Bio-Rad Assay Id	Amplicon Context Sequence	Amplicon Length	Efficiency
Actb	Actin, cytoplasmic 1 Actin, cytoplasmic 1, N-terminally processed	mRNA expression increases following axon injury; may play a role in acceleration of axonal outgrowth	NM_031144	ENSRNOG00000034254	qRnoCID0056984	GTACAACCTTCTTGCAGCTCCTCCGTCGCCGGTCCACACCCGCCACCAGTTCGCCATGGATGACGATATCGCTG	74	97
Apcs	Serum amyloid P-component	a component of amyloid P	NM_017170	ENSRNOG00000009086	qRnoCED0007176	AGTAGCTCATTGTCTCTGCTGTTGACACTGTAGGAGAAAAGACTCTGAGAGCGGGAAAGGTCACTGTAGGCTCGGAAACA	80	100
Atf3	Cyclic AMP-dependent transcription factor ATF-3	expression is associated with neuronal injury	NM_012912	ENSRNOG00000003745	qRnoCED0053089	GTTGTTGATGGTGACTGACTCCAGCGCAGAGGACATCCGATGGCAAAGGTGCTTGTTCTGGATGGCGAATCTCAGCTCTTCCTTG	85	91
B2m	Beta-2-microglobulin	mouse homolog plays a role in the assembly and surface expression of MHC class I molecules	Not Available	ENSRNOG00000017123	qRnoCED0056999	GTGTACTCTCGCCATCCACCGGAGAATGGGAAGCCCAACTTCCTCAACTGCTACGTGTCTCAGTTCCACCCACCTCAAATAGAAATTGAGCTACTGAAGAATGGAA	106	95
Bax	Apoptosis regulator BAX	Bcl2-related gene; involved in the regulation of apoptotic cell death	Not Available	ENSRNOG00000020876	qRnoCED0002625	GTGGACCTGAGGTTTATTGGCACCTCCCCCAGGCCACCATTCCCACCCCTCCCAATAATTACAAAAGTAAGAAAAATGCCTTTCCCCGTTCCCCATTCATCCCAGGAAAAATGTCATAATTTATGAAGAAAAGACACAGTCCAAGGCAGCAGGAAGCCTCAGCCCATCTTCTTCCAGATGGTGAGTG	187	97
Bcl2	Apoptosis regulator Bcl-2	an anti-apoptotic protein; involved in inhibiting cell death in many different cell types.	NM_016993	ENSRNOG00000002791	qRnoCED0006419	TGTGACAGCTTATAATGGATGTACTTCATCACGATCTCCCGGTTATCATACCCTGTTCTCCCGGCTTGCGCCATCCTTCCGGGGAAAGAAGCTGCAGGTACCA	103	96
C3	Complement C3 precursor	putative complement component C3; likely involved in innate immune response	Not Available	ENSRNOG00000046834	qRnoCED0001280	CTGGTGGTGAAAGGTGACCCAAGAGATAACCGACAGCCCGCGCCTGGGCATCAAACGACACTAAGGATCGAGGGGAACCAGGGGGCCCGAGTGGGGCTAGTGGCTGTGGACAAGG	115	95
C5ar1	C5a anaphylatoxin chemotactic receptor	binds complement component 5 and induces Ca+2 release from intracellular stores	NM_053619	ENSRNOG00000047800	qRnoCED0002472	GAACACGGCCAAGTAGATAATAAGGGCTGCAATGTCCCCAGGTTCCATCTTCGGAATGTAAACGCCATCTGCAGGCATGTCAGGATTTGGGGTTCCATCAGAGTAATCATAGGTGA	116	97
Camp	Cathelicidin antimicrobial peptide precursor	The protein plays an important role in innate immunity defense against viruses. In addition to its antibacterial, antifungal, and antiviral activities, the encoded	NM_001100724	ENSRNOG00000020733	qRnoCED0003664	GACTGCTGGTTGAAGTCATCCACAGCACGGAGTACAGCCTCCCTGTAGCTGAGGGTCTGGGAAACGGCTAGAGGCAACCCAAGGCCCAGGAGCAATAGCAGTGACAGTG	109	100

		protein functions in cell chemotaxis, immune mediator induction, and inflammatory response regulation.						
Casp1	Caspase-1	apoptotic protease that may play a role in inflammation and apoptosis in male sex organs	NM_012762	ENSRNOG00000007372	qRnoCED0004262	ACTTCTGACAGTACCTTCCTTGTATTCATGTCTCATGGTCTCCAGGAGGGAATATGTGGGATCACATACTCTAATGAAGTTGCA	84	100
Casp3	Caspase-3 Caspase-3 subunit p17 Caspase-3 subunit p12	apoptotic cysteine-aspartic acid protease that may play a role in neuronal cell death regulation and other apoptotic processes	NM_012922	ENSRNOG00000010475	qRnoCID0006430	CTGCTGTCCAGATATATTCCAGAGTCCATCGACTTGCTTCCATGGATAGTCTTTGTTTCAAAATTATTAATGGATTTTGAATCCACGGAGGTTTCGTTGTTGTCCATGGTCACTT	115	100
Casp8	Caspase-8	member of the cysteine-aspartic acid protease (caspase) familythat mediates the terminal stage of apoptosis; involved in apoptosis induced by Fas and other stimuli	Not Available	ENSRNOG00000012331	qRnoCED0003325	CTGTGCTTGGACCACATCCCGCAGAAGAAGCAGGAGTCCATCAATGATGTCCTGGTGCTAT	61	98
Casp9	Caspase-9	plays a role in initiation and progression of apoptosis	NM_031632	ENSRNOG00000012944	qRnoCID0006496	CGGTGGACATTGGTTCTGGCAGAGCTCATGATGTCTGTACTCCAGGGAAGATCGAGAGACATGCAG	66	98
Ccl12	Chemokine (C-C motif) ligand 12 precursor	This chemokine is found predominantly in lymph nodes and thymus und er normal conditions, and its expression can be hugely induced in macrophages	Not Available	ENSRNOG00000029768	qRnoCED0006489	AGTCACCTGCTGTTATAATGTCGCTAAGCAGAAGATCCACATTCGGAGGCTAAAGAGCTACAGGAAAATCACAAGCAGCCAGTG	84	101
Ccl3	C-C motif chemokine 3	mediates monocyte and neutrophil chemotaxis; may play a role in the pathogenesis of acute lung injury	NM_013025	ENSRNOG00000011205	qRnoCED0002216	AGGTGGCAGGAATGTTCTGGGGCTCAAGCCCCTGCTCTACACGGGGCCCACGGAGGTTTGGGGGTTCCTTGCTGCCTCTAATCTCAGGCATTTAGTTCCAGCTCAGTGATGT	112	96
Ccl5	C-C motif chemokine 5 precursor	may play a role in cellular response to viral infection in testicular somatic cells	NM_031116	ENSRNOG00000010906	qRnoCID0008572	GGTTCCTTCGAGTGACAAAGACGACTGCAAGGTTGGAGCACTTGCTGCTGGTGTAAAAATACTCCTTCACGTGGG	75	99
Ccr3	C-C chemokine receptor type 3	chemokine receptor; may have a role in inflammation and HIV infection	NM_053958	ENSRNOG00000006736	qRnoCED0055609	GGATTCACTATGTTCTGTGGAATGAGTGGGGTTTTGGCCACTGCATGTGTAAAATGCTCTCTGGGCTTTATTACCTGGCCTTGTACAGCGAGATCTTTTTCATCATCCTGCTGACAAT	118	92
Ccr4	C-C chemokine receptor type 4	mouse homolog is a chemokine receptor that binds both macrophage inflammatory protein-1 alpha and RANTES	NM_133532	ENSRNOG00000010315	qRnoCED0005644	ACTTGAGCCTCTTGTACTTGAACAGCACCAGAACCACAACAGAATTCCCAAACAGACCCAACAGAAAGACCAAGGAGTAGAGAGG	85	99

Ccr5	C-C chemokine receptor type 5	G protein-coupled receptor; involved in regulation of leukocyte activation and migration	NM_053960	ENSRNOG00000049115	qRnoCED0003494	AATGCAGGTGACAGAGACTCTTGGGATGACACACTGCTGCCTCAACCCTGTCATCTATGCCTTTGTTGGGGAGAAGTTCCGGAATTATCTCTCTGTGTTCTTCCGA	106	98
Ccr6	C-C chemokine receptor type 6	The gene is preferentially expressed by immature dendritic cells and memory T cells. The ligand of this receptor is macrophage inflammatory protein 3 alpha (MIP-3 alpha). This receptor has been shown to be important for B-lineage maturation and antigen-driven B-cell differentiation, and it may regulate the migration and recruitment of dentritic and T cells during inflammatory and immunological responses.	Not Available	ENSRNOG00000012964	qRnoCED0052490	GTCAGTGGCATGAGTAACTGCCCAGAATGGTAGGGTGAGGACAAAGAGTATGTCTGTGATGGCCATGTTCAATAGGTAGACGTCAGTCATGG	92	90
Ccr8	Protein Ccr8	this receptor may contribute to the proper positioning of activated T cells within the antigenic challenge sites and specialized areas of lymphoid tissues.	Not Available	ENSRNOG00000026759	qRnoCED0001453	GTATCTGGCCGTCTTATACTGCATCTTGTTTGTGCTGGGCCTTCTGGGAAACAGCCTGGTCATCTTGGTCCTTGTGGCCTGCAAGAAACTGAGGAGTATCACGGACGTCTACC	113	98
Cd14	Monocyte differentiation antigen CD14	component of the lipopolysaccharide receptor complex; mediates LPS-induced neuroinflammation and inflammatory response	Not Available	ENSRNOG00000017819	qRnoCED0008863	AAGTTGAGTGAGTGTGCTTGGGCAATACTTAGTACCTTGAGTCCAGGCTTTAGCCACTGCTGCAGTTCTGCGAGCCAGGTAT	82	101
Cd1d1	Antigen-presenting glycoprotein CD1d precursor	an antigen-presenting protein distinct from the major histocompatibility complex	NM_017079	ENSRNOG00000016451	qRnoCED0007785	GTGCTGTCTAGTTCAGTACAAATCCCACGAGACACTGCTCACAGCTTGTTTCTGGCAGGTACACACAT	68	103
Cd3d	T-cell surface glycoprotein CD3 delta chain	putative component of the CD3 T-cell receptor	Not Available	ENSRNOG00000015994	qRnoCID0003978	TGTGGAAGATGGAGCACTATGGAATTCTGGTTAGTCTGCTACTGGCTACTGTTCTCCCCCAAGGGAGCCCCTTCAAGATAGAAGTGGTTGAATATGAGGA	100	95
Cd4	T-cell surface glycoprotein CD4	MHC class II binding protein that may be a candidate gene for collagen-induced arthritis; human CD4 is a receptor required for HIV infection	NM_012705	ENSRNOG00000016294	qRnoCED0002294	AGATGCCACTGTCCTGAATCCTTAGGCTGTGCGTGGAGAAAGCTTTGGAGTCCTTGACAATGTT	64	101

Cd40	Tumor necrosis factor receptor superfamily member 5 precursor	may mediate chronic inflammation including arteriosclerosis; interacts with ligand CD154 and plays a crucial role in humoral and cellular immunity, and in T-cell-mediated inflammatory responses	NM_134360	ENSRNOG00000018488	qRnoCID0003897	CTGATCTCGCTCTGCAATGCTGCCTTTGCCTCAGCTGTGCGCGCTCTGGGGCTGCTTGTTGACAGCGGTCCATCTAGGACAGTGTGTTACGTGCAGTGACAAACAGTACCTCCAAGGTGG	120	91
Cd40lg	CD40 ligand	CD40 ligand	NM_053353	ENSRNOG00000000871	qRnoCID0009370	TTGCAGCACATGTTGTAAGTGAGGCCAACAGTAATGCAGCATCTGTTCTTCAGTGGGCGAAGAAAGGATATTATACCATGAAAAGCAACTTGGTAGTGCTG	101	99
Cd80	T-lymphocyte activation antigen CD80	cell surface protein that is part of the immune response; may be involved in induction of experimental autoimmune anterior uveitis	NM_012926	ENSRNOG00000001527	qRnoCID0009234	GTCTCAGGTTCATTCATCTCTTTGTGCTGCTGCTGGTTGGTCTTTTCCAGATATCTTCAGGTATTGTCGGCCAAGTGTCCAAGTCGGTGAGAGAAAAGGCATTGCTGTCCTGTGATTACA	120	97
Cd86	T-lymphocyte activation antigen CD86 precursor	plays a role in T-cell activation and proliferation	NM_020081	ENSRNOG00000038835	qRnoCED0008398	AACTAATGAGTATGGCGACAACATGCAGATATCACAAGACAATGTCACAAAGCTGTTCAGTGTCTCCATCAGCCTATCTCTTCC	84	96
Cd8a	T-cell surface glycoprotein CD8 alpha chain	Increased expression on mast cells is induced by nitric oxide; may play a role in inflammatory response.	Not Available	ENSRNOG00000007178	qRnoCED0001354	GGATGCTCTTGGCTCTTCCGGAACTCCAGCTCCGAACTCCTCCAGCCCACCTTCATCATCTATGTATCTTCATCCCGGAGCAAGCTGAACGATATACTGGATCCGA	106	94
Ckm	Creatine kinase M-type	Catalyzes regeneration of ATP; expression is induced by p53.	NM_012530	ENSRNOG00000016837	qRnoCED0005864	CAAGTCGCAGGAGGAGTACCCAGACCTCAGCAAACACAACAACCACATGGCCAAGGTGCTGACTCCTGACCTCTACAATAAGCTTCGAGACAAGGAG	97	101
Crp	C-reactive protein	Glycoprotein of the acute phase response.	NM_017096	ENSRNOG00000000053	qRnoCED0002177	AGGAGCAGGACTCGTATGGCGGTGGCTTTGACGCGAATCAGTCTTTGGTGGGAGACATTGGAGATGTGAACATGTGGGACTTTGTGCTATCTCCAGAACAG	101	101
Csf1	Macrophage colony-stimulating factor 1 Processed macrophage colony-stimulating factor 1	Plays a role in macrophage formation; involved in osteoclastogenesis and endochondral ossification.	Not Available	ENSRNOG00000018659	qRnoCID0004474	AGCTGTTCCTGGTCTACAAATTTATATTCGATCAGGCATGCAGTCTCCATTTGGCTGTCGATCAACTGCTGCAAAATCTGTAGGTGTCCATTCC	94	100
Csf2	Granulocyte-macrophage colony-stimulating factor	Plays a role in alveolar epithelial fluid transport.	Not Available	ENSRNOG00000026805	qRnoCED0004359	GCAGTTCGTCTGGTAGTGGCTGGCTATCATGGTCAAGGCGCCATTGAGTTTGGTGAGGTTGCCCCGTAGACCCTGCTTGTATAGCTTCAGGC	92	103
Csf3	Granulocyte colony-stimulating factor precursor	Putative hematopoetic growth factor for neutrophils; used as a treatment for cyclic hematopoesis in humans and dogs.	Not Available	ENSRNOG00000008525	qRnoCED0001885	GCACTATGGTCAGGACAAGAGGCCATTCCCCTGCTCACCGTCAGCTCTCTGCCACCATCCCTGCCTTTGCCCCGAAGCTTTCTGCTTAAGTCCTTGGAGCAAGTGAGGAAGATTCAG	117	98

Cx3cl1	Fractalkine precursor	Chemokine that binds its receptor CX3CR1; induces chemotaxis and increased intracellular calcium levels in microglia.	NM_134455	ENSRNOG00000016326	qRnoCED0007474	GACAGAACGAGTTGTTGGTGGTGATGGTGGTGATGGCTCTGTACACGGGAGAGAGAGACCGGGATAGATAGCACCGCAGC	80	102
Cxcl1	Growth-regulated alpha protein precursor	Acts as a neutrophil chemoattractant; may play a role in acute phase inflammatory response.	NM_030845	ENSRNOG00000002802	qRnoCED0003672	TCACACATTTCCTCACCCTAACACAAAACACGATCCCAGACTCTCATCTCTCCGCCCTTCTTCCCGCTCAACACCTTCTAGCAC	84	100
Cxcl10	C-X-C motif chemokine 10	Induces DNA synthesis, cell proliferation, and cell migration in vascular smooth muscle cells; may play a role in vascular remodeling.	NM_139089	ENSRNOG00000022256	qRnoCED0009075	TCTAAGAGCTGGTCCGAATCTTCCCTCAGGCAGCTATGACGGCTCTCCTAGCTCTGTTCTGTAAGCTATGTGCAGGTACTAATCTCTTCAGCATGTGCC	99	97
Cxcl2	C-X-C motif chemokine 2	Chemokine involved in the pulmonary inflammatory response	NM_053647	ENSRNOG00000002792	qRnoCED0003624	GCTGACTGAACACATTGAACATTATTACAATAAACTTCAACATATTAAAATGACCTCTTAAGATACTACAGTGAGCTGGCCAATGCATATCTTTAAATATCA	102	99
Cxcr3	C-X-C chemokine receptor type 3	Binds interferon-inducible protein-10 and induces intracellular calcium mobilization; increased expression occurs in response to focal stroke	NM_053415	ENSRNOG00000003305	qRnoCED0006002	ACTAGCCTCATAGCTCGAAAGCGCCTCTGGCCTCTGGAGACCAGCAGCACAGCCAGGATATGGGCATAGCAGTAGGCCATGACTA	85	98
Ddx58	Probable ATP-dependent RNA helicase DDX58	This gene encodes a protein containing RNA helicase-DEAD box protein motifs and a caspase recruitment domain (CARD). It is involved in viral double-stranded (ds) RNA recognition and the regulation of the antiviral innate immune response.	Not Available	ENSRNOG00000006384	qRnoCID0007853	GCAAGCTCCAGTTGGTAATTTCTTGGTTTCAATGGGCTGTGTGAATTATTAGGAGACGCTTCTGAAGGAGGGCCGGGATTCTGACTGAGATTCTGACACTCCGGTTC	107	102
Eng	Endoglin	A major glycoprotein of the vascular endothelium and a component of the transforming growth factor beta receptor complex and it binds to the beta1 and beta3 peptides with high affinity.	Not Available	ENSRNOG00000050190	qRnoCID0003750	CTACAAGACTGTCTCCATGCGCCTGAACATCGTCAGTCCTGACCTGTCTGGCAAAGGCCTCGTCCTGCCCTCTGTACTGGGCATCACCTTTGGTGCCTTCCTCATTGGGGCCCTGCTCACAGCTGCACTCTGGTACATCTATTCTCACACACGTGAGTATCCCAAGCCTCTCCACCCCCTTCCTACAGTGAGTGCTCA	198	98
Fadd	Protein FADD	An important adaptor molecule in apoptosis signaling; may mediate hepatocyte death.	NM_152937	ENSRNOG00000047035	qRnoCED0007204	GTTCTCCTTCTCGACATTCTTCCAGACTCGCAGAGTCTCCCTTACCCGATCACTCAGGCTTCGGGGGTACCTCTCCTCAATGCCATCAATCTTGGCCTCAGACACCTTCAGCTCA	115	103

Faslg	Tumor necrosis factor ligand superfamily member 6 Tumor necrosis factor ligand superfamily member 6, membrane form Tumor necrosis factor ligand superfamily member 6, soluble form ADAM10-processed FasL form FasL intracellular domain	A ligand that binds the Fas receptor; plays a role in induction of apoptosis.	NM_012908	ENSRNOG00000002978	qRnoCED0003046	TGCCAGTTCCTTCTGTAGATGAAAGAGTTGATACATTCCTAACCCCATTCCAACCAGAGCCACCAGCACCATGAAAAATATCACCGGTAGCCACA	95	103
Fos	Proto-oncogene c-Fos	An immediate early gene encoding a nuclear protein involved in signal transduction.	NM_022197	ENSRNOG00000008015	qRnoCED0002247	AGCTCTCAGGTGTCACTACAAACAATACACTCCATGCGGTTGCTTTTGATTTTTTGTTTTGTTTTTTTTTTGTTTTGTTTTTTTGTTTTTTTGCTACATCTCCGGAAGA	109	99
Foxp3	Forkhead box protein P3	The protein encoded by this gene is a member of the forkhead/winged-helix family of transcriptional regulators.	NM_001108250	ENSRNOG00000011702	qRnoCED0052464	GTAAAGGGTGGTCTTACAGGGGCATAGAGTAGTGGAAAAGGGGGCAATAGCTTGCTGTTATCTCTGAGGTCCCTTTTGTCTTATCAGGATGCTAAGAAGGATGATGCTG	109	99
Gapdh	Glyceraldehyde-3-phosphate dehydrogenase	A key glycolytic enzyme that converts D-glyceraldehyde 3-phosphate (G3P) into 3-phospho-D-glyceroyl phosphate.	NM_017008	ENSRNOG00000018630	qRnoCID0057018	TTTGTCACAAGAGAAGGCAGCCCTGGTAACCAGGCGTCCGATACGGCCAAATCCGTTCACACCGACCTTCACCATCTTGTCTATGAGACGAGGCTGGCACTGCACAAGAAGATGC	115	96
Gata3	GATA binding protein 3	Transcription factor which is responsible for Th2 commitment during anterior chamber associated immune deviation development.	Not Available	ENSRNOG00000019336	qRnoCID0008066	ATGTAAGTCGAGGCCCAAGGCACGATCCAGCACAGAAGGCAGGGAGTGTGTGAACTGCGGGGCAACCTCTACCCCACTGTGGCGGCGAGATGGTACTGGGCACTA	105	93
Havcr1	Hepatitis A virus cellular receptor 1 homolog precursor	Member of the mucosal addressin cell adhesion molecule family; may be involved in restoration of the morphological integrity and function to postischemic kidney.	NM_173149	ENSRNOG00000007243	qRnoCID0004444	AACACCATGGTTCAACTTCAAGTCTTCATTTCAGGCCTCCTGCTGCTTCTTCCAGGCTCTGTAGATTCTTATGAAGTAGTGAAGG	85	97
Hprt1	Hypoxanthine-guanine phosphoribosyltransferase	Catalyzes the conversion of IMP and diphosphate to hypoxanthine and 5-phospho-alpha-D-ribose 1-diphosphate.	NM_012583	ENSRNOG00000031367	qRnoCED0057020	GTAGATGGCCACAGGACTAGAACGTCTGCTAGTTCTTTACTGGCCACATCAACAGGACTCTTGTAGATTCAACTTGCCG	79	98

Hsp90ab1	Heat shock protein HSP 90-beta	Displays chaperone activity; binds to peptides and facilitates their appropriate transport and/or secondary structure.	NM_001004082	ENSRNOG00000019834	qRnoCID0003832	CTACTACTCGGCTTTCTCGTCAAGATGCCTGAGGAAGTGCACCATGGAGAGGAAGAGGTGGAGACCTTCGCCTTTCAGGCAGAAATTGCCCAGCTGATGTCCCTCATCATCAACAC	116	99
Hspa2	Heat shock-related 70 kDa protein 2	Involved in repeated binding and release of substrate.	NM_021863	ENSRNOG00000006472	qRnoCED0002766	CCTATACCTACAACATCAAGCAGACGGTGGAAGACGAGAAACTGAGGGGCAAGATTAGCGAGCAGGACAAAAACAAGATTCTCGACAAGTGTCAGG	96	98
Hspa4	Heat shock 70 kDa protein 4	May have an important role following forebrain ischemia and may allow astrocytes to protect neurons.	NM_153629	ENSRNOG00000016596	qRnoCID0051612	ATCACTGACCGTCGTTCTGCATCAGTGTAGAAACTAGGAACCGAAACAACACAGTCTACAACAGGCTTCTTAAGAACACTTTCTGCTGTCTCCTTCAGTTTTGACAAGAGCATGGCAGTC	120	91
Hspb1	Heat shock protein beta-1	Putative heat shock protein; human homolog may suppress polyglutamine-mediated cell death and mutations in human gene are associated with various neuropathies and some forms of Charcot-Marie-Tooth disease.	NM_031970	ENSRNOG00000023546	qRnoCED0001063	GCTGAGTTGCCGGTTGAGCGCCCGGCTGAAGGCGGGCGCGGCCAGGGTCACTGCTGCGGGGCCCTCGGCGGTCGCGGCGGGCAGAGGGCGCACATAGCCGGGCCAACCAGCGGAGCTGAACCACTGAGACCACTCATCGGGAAACCGAGGCACCCCGAAAGCTTGATCGAAGA	173	93
Icam1	Intercellular adhesion molecule 1	Cell adhesion molecule; ligand for leukocyte adhesion molecule LFA-1	NM_012967	ENSRNOG00000020679	qRnoCED0005284	GTTGGTGTGTTGGTACTGATCATTGCGGGCTTCGTGATCGTGGCGTCCATTTACACCTATTACCGCCAGAGGAAGATCAGGATATACAAGTTAC	94	100
Ifna1	Interferon alpha-1 precursor	A cytokine of the type I interferon family that is produced in response to viral infection as a key part of the innate immune response with potent antiviral, antiproliferative and immunomodulatory properties.	NM_001014786	ENSRNOG00000031046	qRnoCED0001081	TATTCCCTCACAGCCAGTAGGGAGTCTTCCTGGGTCAGGGGAGATTCCTGCACCCCTACCTGCTGCATCAGACAGGCTTGCAGACCACTCAGCTGCTGCTGGAGGTCATTACAGAATGAGTCTAGGAGGG	130	91
Ifnar1	Interferon alpha/beta receptor 1 isoform 2	A type I membrane protein that forms one of the two chains of a receptor for interferons alpha and beta. Binding and activation of the receptor stimulates Janus protein kinases, which in turn phosphorylate several proteins, including STAT1 and STAT2.	Not Available	ENSRNOG00000028594	qRnoCED0005522	AACCGCCTAATCCTTGTTCACTGATAGTTCTTGGGGAAGCTGAGCCGTCTAAGAACCGTGTAAAAGACAAGAGTGAGGTGCT	82	96
Ifnb1	Interferon beta	Suppresses the growth of rat glioma cells	NM_019127	ENSRNOG00000006268	qRnoCED0002914	GACATTCTGGAGCATCACTTGAATGGCAAAGGCAGTGTAACTCTTCTCCATCTGTGACGGGTGCATCACCTCCATAGGGATCTTGAAGTCCGTCCTGTA	99	93

Ifng	Interferon gamma	An immune molecule produced by T lymphocytes in response to mitogens or antigens	NM_138880	ENSRNOG00000007468	qRnoCID0006848	TCACTAACTTCTTCAGCAACAGTAAAGCAAAAAAGGATGCATTCATGAGCATCGCCAAGTTCGAGGTGAACAACCCACAGATCCAGCACAAAGCTGTCAATGAACTCATCAGAGTGATTC	120	97
Ifngr1	Interferon gamma receptor 1 precursor	Mouse homolog is a receptor that binds IFN-gamma	NM_053783	ENSRNOG00000012074	qRnoCID0001888	TACATTGGAAGCACCAGAACGTGTCGCAGGCTGCCGTCTTCACTGTACAGGTAAAGATGTATCCGGATAACTGGACTGATGCCTG	85	99
Il1a	Interleukin-1 alpha precursor	A pleiotropic cytokine involved in various immune responses, inflammatory processes, and hematopoiesis. This cytokine is produced by monocytes and macrophages as a proprotein, which is proteolytically processed and released in response to cell injury, and thus induces apoptosis.	NM_017019	ENSRNOG00000004575	qRnoCID0002952	ATGACCTGGAGGCCATAGCCCATGATTTAGAAGAGACCATCCAACCCAGATCAGCACCTCACAGCTTCCAGAATAATTTGAGATACAAATTGATAAGGATCGTCAAGCAGG	111	102
Il1b	Interleukin-1 beta precursor	Produced by activated macrophages as a proprotein, which is proteolytically processed to its active form by caspase 1 (CASP1/ICE). This cytokine is an important mediator of the inflammatory response, and is involved in a variety of cellular activities, including cell proliferation, differentiation, and apoptosis.	Not Available	ENSRNOG00000004649	qRnoCID0004680	GACAGAACATAAGCCAACAAGTGGTATTCTCCATGAGCTTTGTACAAGGAGAGACAAGCAACGACAAAATCCCTGTGGCCTTGGGCCTCAAGGGGAAGAATCTATACCTGTCCTGTGTGA	120	98
Il1r1	Interleukin-1 receptor type 1	A receptor for interleukin-1; involved in inflammatory response signaling.	Not Available	ENSRNOG00000014504	qRnoCID0007565	GGACAGACCTGTGATTATGAGCCCACGGAATGAGACGATGGAAGCTGACCCAGGATCCACGATACAACTGATCTGCAACGTC	82	98
Il2	Interleukin-2	A cytokine produced by T-cells in response to antigen or mitogen stimulation.	Not Available	ENSRNOG00000017348	qRnoCED0006493	AGTCATTGTTGAGATGATGCTTTGACAGATGGCTATCCATCTCCTCAGAAATTCCACCACAGTTGCTGGCTCATCATCGAATTGGCACTCAA	92	97
Il4	Interleukin-4	Th2-type cytokine; may be involved in inflammatory response in eosinophils.	NM_201270	ENSRNOG00000007624	qRnoCID0002254	GAGCGTGGACTCATTCACGGTGCAGCTTCTCAGTGAGTTCAGACCGCTGACACCTCTACAGAGTTTCCTCAGTTCACCGAGAACCCCAGACTTGTTCTTCAAGCACGGA	109	98
Il5	Interleukin-5	A cytokine with B-cell growth factor activity.	Not Available	ENSRNOG00000008111	qRnoCED0001585	GGATGCTTCTGTGCTTGAACGTTCTAACTCTCAGCTGTGTCTGGGCCATTGCTATGGAGATCCCCATGAGCA	102	99

						CAGTGGTGAAAGAGACCTTGATACAGCTGT		
Il6	Interleukin-6	A cytokine involved in development and possibly in neurodegenerative processes	NM_012589	ENSRNOG00000010278	qRnoCID0053166	CAGACCAGTATATACCACTTCACAAGTCGGAGGCTTAATTACATATGTTCTCAGGGAGATCTTGGAAATGAGAAAAGAGTTGTGCAATGGCAATTCTGATTGTATGAACAGCGATGATGC	120	94
Il10	Interleukin-10	A cytokine produced primarily by monocytes and to a lesser extent by lymphocytes. This cytokine has pleiotropic effects in immunoregulation and inflammation. It down-regulates the expression of Th1 cytokines, MHC class II Ags, and costimulatory molecules on macrophages. It also enhances B cell survival, proliferation, and antibody production.	NM_012854	ENSRNOG00000004647	qRnoCID0005930	CCTTCAGTCAAGTGAAGACTTTCTTTCAAAAGAAGGACCAGCTGGACAACATACTGCTGACAGATTCCTTACTGCAGGAC	80	98
Il12a	Interleukin-12 subunit alpha precursor	A subunit of a cytokine that acts on T and natural killer cells, and has a broad array of biological activities; required for the T-cell-independent induction of interferon (IFN)-gamma, and is important for the differentiation of both Th1 and Th2 cells.	Not Available	ENSRNOG00000009468	qRnoCID0006153	CTGCCAAGTGTCTTAACCAGTCCCAAAACCTGCTGAAGACCACGGACGACATGGTGAGGACGGCCAGAGAAAAATTGAAACATTACTCTTGCACTGCTG	99	98
Il13	Interleukin-13	A cytokine involved in several stages of B-cell maturation and differentiation and also down-regulates macrophage activity, thereby inhibits the production of pro-inflammatory cytokines and chemokines.	Not Available	ENSRNOG00000007652	qRnoCID0008414	CATTGCAACTGGAGATGTTGGTCAGGGATTCCAGGGCTGCACAGAACCCGCCAGCTGTCAGGTCCACGCTCCATACCATGCTGCTGTTGCACAGGGAAGTCTTCTGGTCTTGTGTGATGT	120	101
Il18	Interleukin-18	A cytokine that is implicated in the injury of different organs, and in potentially fatal conditions characterized by a cytokine storm.	NM_019165	ENSRNOG00000009848	qRnoCID0003876	TCGAGGACATGCCTGATATCGACCGAACAGCCAACGAATCCCAGACCAGACTGATAATATATATGTACAAAGATAGTGAAGTAAGAGGACTGGCTGT	97	98
Il23a	Interleukin-23 subunit alpha	Mouse homolog is a proinflammatory cytokine; high levels of expression may be	Not Available	ENSRNOG00000003254	qRnoCED0006916	AGTCAGTCCATGTTGGTAGTTCTGTTAGTTCTTAGTGCTGGGTTCTGTTAGAACTGAAGGACTAAGCAGGCAGTTACAGAGCTTCCGTCTG	91	101

		associated with rheumatoid arthritis, psoriasis, and multiple sclerosis.						
Irak1	Interleukin-1 receptor-associated kinase 1	This gene encodes the interleukin-1 receptor-associated kinase 1, one of two putative serine/threonine kinases that become associated with the interleukin-1 receptor (IL1R) upon stimulation.	NM_001127555	ENSRNOG00000037263	qRnoCID0003837	TGGCTTCTACTGCCTTGTTTATGGCTTCCTGCCCAATGGCTCCTTAGAGGATCAGCTTCACCTTCAGACCCAAGCCTGCTCCCCACTTTCCTGGCCTCAACGACTGGACATTCTT	115	101
Irf3	Interferon regulatory factor 3	The protein plays an important role in the innate immune response against DNA and RNA viruses.	Not Available	ENSRNOG00000043388	qRnoCED0006338	TCAGACCTGCCTATTGCTTCTGATCCTTCTCAACCACCACTAAGCCCCATTGTAAACAACTTCCC	65	102
Irf7	Interferon regulatory factor 7	Play a role in the transcriptional activation of virus-inducible cellular genes, including interferon beta chain genes.	Not Available	ENSRNOG00000017414	qRnoCED0005624	AAGGTCCACTAGAGATGACATATAGCCAAGGAATAAGCCTGAGCCAGGGCAGCAGTGGTTCTGAACTCTATTGGAAG	77	99
Itga1	Integrin alpha-1	Alpha subunit of the integrin molecule; involved in binding laminin and collagen	Not Available	ENSRNOG00000012080	qRnoCID0005310	TTGTCGATGTCAATTGTTGTTAAGACACTACCAAAGTAGGAACCAATCTGCTCTCCGCCGAGTGTCTGCAGAATGTTGATGTTCCCATCCTCCATCTTGTAGATGACGACCTGG	114	100
Itga2	Protein Itga2	Mouse homolog forms integrin with beta 1 subunit; involved in placental development and matrix assembly and remodeling	Not Available	ENSRNOG00000011877	qRnoCID0006566	GGAATGTGGATGGTTACTGATGCCATGCTCACTGGAGCACTTCCCGCTGTTATCTTGAGAGAGAAGATGAATTCAGGACCA	81	99
Itgam	Integrin alpha-M precursor	Mouse homolog is an integrin alpha subunit that interacts with integrin beta 2 subunit to form Mac-1; involved in immune response	NM_012711	ENSRNOG00000019728	qRnoCID0002800	TGCGTGTCAAGAAGAAGTAGTCAAAAACAAGGATGCTGGGGAGGTCAGAGTCTGCCTCCATGTCCGCAAGAACACCAAGGACAGGCTGCGAGAAGGAGACATCCAGAGCACTGTCACTTA	120	94
Itgb2	Integrin beta 2 precursor	Plays an important role in immune response and defects in this gene cause leukocyte adhesion deficiency.	NM_001037780	ENSRNOG00000001224	qRnoCID0053063	TGCGTCTGACACTCACAGTTTTTCCCAATGTAGCCAGACTCACACCTGCAGATGCCACACTCCATGACTCCCTTGCCTCCACAGAGATTCCTCATCCGACTC	102	97
Jak2	Tyrosine-protein kinase JAK2	Associated with receptors for erythropoietin, prolactin, growth hormones and interleukin-3; phosphorylated when activated by cytokines	Not Available	ENSRNOG00000015547	qRnoCID0004742	GATGAGTCAACCAGGCATAATATACTCTACAGAATAAGGTTTTACTTTCCTCATTGGTACTGCAGTGGCAGCAACAGAACCTATAGGTACGGAGTATCTCGTG	103	102

Jun	Transcription factor AP-1	Transcription factor; acts as a protooncogene.	Not Available	ENSRNOG00000026293	qRnoCED0006525	ATTCTGGCTATGCAGTTCAGCTAGGGCGCGCACGAAGCCTTCGGCGAAGCCCTCCTGCTCGTCGGTCACGTTCTTGGGGCACAAGAACTGAGTGGGGGTCGGTGTAGTGGTGATGTGCC	119	101
Lbp	Lipopolysaccharide-binding protein precursor	Binds lipopolysaccharide on outer membrane of gram negative bacteria; involved in immune response.	NM_017208	ENSRNOG00000014532	qRnoCID0007468	TGTCGATATCCGCTGTGACTGGCAGAGTTTGGAGATAAGGCTGCAGATCAGAGGTCACAGACTTCTGGATCATCTCACAA	80	97
Lcn2	Neutrophil gelatinase-associated lipocalin	Mouse homolog plays a role in IL3 withdrawal-induced apoptosis.	NM_130741	ENSRNOG00000013973	qRnoCED0001706	CTGACGAGGATGGAAGTGACGTTGTAGCTATTGTCTTCCTGTAGCTCATAGATGGTGCTGTACATGGT	68	99
Lyz2	Lysozyme C-1 precursor	Widely expressed gene, may mediate analgesic, anti-inflammatory, hemostatic, anti-allergy, anticancer, and antibiotic functions.	NM_012771	ENSRNOG00000005825	qRnoCID0051084	GGGATCCCTCACAACTCTCTTCGCACATTGTATGGCTTGAGTGATGTCATCCTGCAGCAGAGCGCTGCAGGGTATCCCACAGGCGTTCTTTGCTCTTGGGGTTTTGCCGTCATTACACCAGTATCGGCTATTGATCTGAAATATCCCATAGTCGGTGCTTTGGTCTCCAGGGTTGTAGTTTCTGGCTTGTGTGTTAT	197	88
Mapk1	Mitogen-activated protein kinase 1	This kinase is involved in intracellular signaling; component of Mapk signaling pathway.	NM_053842	ENSRNOG00000001849	qRnoCID0003206	GCGTCCAGTCCTCTGAGCCCTTGTCCAATTTAAGATCTGTATCCTGGCTGGAATCGAGCAGTCTCTTCAAAAATGAGTTCTTTGAGCTTCTCCTTAGGTAAGTCGTCCAG	110	103
Mapk14	Mitogen-activated protein kinase 14	Mitogen-activated protein kinase; involved in intracellular signalling, inhibition of apoptosis and gene activation.	NM_031020	ENSRNOG00000000513	qRnoCID0005775	ACCAGCTTCAGCAGATAATGCGTCTGACGGGGACACCCCCTGCTTATCTCATTAACAGGATGCCAAGCCATGAGGCAAGAAACTACATTCAGTCTCTGGC	100	95
Mapk3	Mitogen-activated protein kinase 3	This kinase is involved in intracellular signalling; component of Mapk signalling pathway.	NM_017347	ENSRNOG00000019601	qRnoCID0002469	CTGGAAGCCATGAGAGATGTTTACATTGTTCAGGACCTCATGGAGACGGACCTGTACAAGCTGCTAAAGAGCCAGCAGCTGAGCAATGACCACATCTGCTACT	103	100
Mapk8	Mitogen-activated protein kinase 8	This kinase play a key role in T cell proliferation, apoptosis and differentiation.	Not Available	ENSRNOG00000020155	qRnoCID0006206	CATGACCTCCTTGTATATCAGTTCTTTCCACTCCTCTATTGTGTGCTCCCTTTCATCTAACTGCTTGTCAGGGATCTTTGGTGGTGGGGCCTCTGCTTCTGAAGGATCATACCAGACGT	119	101
Mbl2	Mannose-Binding Lectin 2	An important element in the innate immune system as it recognizes and binds to mannose and N-acetylglucosamine on many microorganisms, including bacteria, yeast, and viruses.	Not Available	ENSRNOG00000011637	qRnoCID0017891	TCCATACGAACAAAGCTTGATCCATGCAGCAACAGAGTCTAAAGCAAACAGCGAAAACCAGCATTATACTCTAGAAAAGCAAAGCGTTCACATTTCAGTTGTGTCTGTTGAGTTCC	116	96
Mmp10	Stromelysin-2 precursor	Enzyme and a transformation-associated protein;	Not Available	ENSRNOG00000032832	qRnoCED0006117	GGATCATCACAGTTCGAGTTTGACCCCAATGCCAGGACGGTGACACACACACTGAAGAGCAACAGCTGGC	113	94

		activates procollagenase and degrades fibronectin and some gelatins				TGTTGTGCTGATTATCATGATGACAAGACATATACAACACTGT		
Mpo	Myeloperoxidase precursor	Myeloperoxidase family member that may contribute to hyperoxia-mediated lung injury via nitration of proteins, resulting in reactive nitrogen species	Not Available	ENSRNOG00000008310	qRnoCED0008317	TGTTCTTAGACACGGTAGTGATGCCAGTGTTGTCACAGATGATGCGGGGCAAGGAGATGGTAGCCAAGG	69	98
Mx2	Interferon-induced GTP-binding protein Mx2	Involved in inhibiting vesicular stomatitis virus	NM_134350	ENSRNOG00000001963	qRnoCED0003396	AGGACATCACTGCCATAGTAGAAGGGGAAGAGATTGTGAGGGAGAAAGAATGTCGCCTATTCACCAAGCTCCGTAAAGAGTTCTTCTTGTGGAGTGAGGAGAT	103	99
Myd88	Myeloid differentiation primary response protein MyD88	An adaptor protein which binds toll-like receptors and links them to other downstream signalling molecules	Not Available	ENSRNOG00000013634	qRnoCED0002859	GCAGACATGGCAAGCAACCCTGGGCCCCGGTTCTGTACGGTGCGCTCGCTTGTTGAGCTTCCTACTTCC	69	100
Nfkb1	Nuclear factor NF-kappa-B p105 subunit Nuclear factor NF-kappa-B p50 subunit	A transcription regulator that is activated by various intra- and extra-cellular stimuli such as cytokines, oxidant-free radicals, ultraviolet irradiation, and bacterial or viral products.	Not Available	ENSRNOG00000023258	qRnoCID0003698	TCACTCTTGGCACAATCTCTAGGCTCGTTTTTAAATTTGGTGTTTATGGTGCCATGGGTGATGCCTGTGTTGGATTTAGTGGCTCCGGGAT	91	92
Nfkbia	NF-kappa-B inhibitor alpha	Inhibitor of NF-kappa-B; binds NF kappa B and retains it in the cytoplasm	NM_001105720	ENSRNOG00000007390	qRnoCED0004928	AATATACAAGTCCACGTTCCTTTGGCCACTTTCCTCTTATAACGTCAGACGCTGGCCTCCAAACACACAGTCATCGTAGG	80	102
Nlrp3	NACHT, LRR and PYD domains-containing protein 3	This protein interacts with the apoptosis-associated speck-like protein PYCARD/ASC, which contains a caspase recruitment domain, and is a member of the NLRP3 inflammasome complex. This complex functions as an upstream activator of NF-kappaB signaling, and it plays a role in the regulation of inflammation, the immune response, and apoptosis.	NM_001191642	ENSRNOG00000003170	qRnoCID0006865	GGAGACTCAGGAGTTCAAGTTTTGTGTGAAAAAATGAAGGACCCACAGTGTAACTTGCAGAAGCTGGGGTTGGTGAATTCCGGCCTTACTTCACTGTGCTGCTC	104	98

Nod2	Nucleotide-binding oligomerization domain-containing protein 2	Primarily expressed in the peripheral blood leukocytes and plays a role in the immune response to intracellular bacterial lipopolysaccharides (LPS) by recognizing the muramyl dipeptide (MDP) derived from them and activating the NFKB protein.	NM_001106172	ENSRNOG00000014124	qRnoCID0005178	AAGCCAGCAACATAGTAACCGAGGGATCTTCTTGAACTCATCTCCCATGCTTGGAGTCAGAGCTCCTCTAGTGACTTGTTCTTCTCCAGCATCA	94	105
Nos2	Nitric Oxide Synthase 2	Cytokine-inducible enzmyme involved in nitric oxide (NO) production	NM_012611	ENSRNOG00000049980	qRnoCID0004849	GATGCTTGTGACTCTTAGGGTCATCCTGTGTTGTTGGGCTGGGAATAGCACCTGGGGTTTTCTCCACGTTGTTGTTAATGTCCTTTTCCTCTTTCAGGTCACCTTGGTA	109	101
Nos3	Nitric oxide synthase, endothelial	Enzyme that synthesizes Nitric oxide from L-arginine	NM_021838	ENSRNOG00000009348	qRnoCID0005021	CCACAGTGATGAGGTTGTCCGGGTGTCTAGATCCATGCAGACAGCCACATCCTCAAGTATGTTGTATCGGTGAGGGTCACACAGGTCCCTCATGCCAATCTCTGAACTCATGTACCA	117	103
Pdgfa	Platelet-derived growth factor subunit A	Alpha chain of platelet derived growth factor; acts as a homodimer or heterodimer with PDGF beta to activate PDGF receptors	NM_012801	ENSRNOG00000001312	qRnoCID0008186	CAGACCATCGGGAGGAGGAGACGGATGTGAGGTGAGATGAGCTGGCTGCCCTCTCCCGGGACACGGATGTACGTGGCGTGTGACATTCCTGAACATACTATGTATGGTGCTTCATTGC	118	99
Pgk1	Phosphoglycerate kinase 1	Kinase enzyme that is important for phosphoprotein glycolysis	NM_053291	ENSRNOG00000002467	qRnoCED0002588	GCTCTCTTCGCTGTATGTAGCCTCTGGTTAGCTTTGTCACTGTTCATGACAGCATGGAAATAACGGTGAGATTCCAGC	78	98
Pink1	Serine/threonine-protein kinase PINK1, mitochondrial	Involved in transferase activity, transferring phosphorus-containing groups and protein tyrosine kinase activity	Not Available	ENSRNOG00000015385	qRnoCED0008437	GCCTGTCCTATCAGATAATCCTCCAGGCGGAAGCCCTGCCAACGTCGTGTGTCCAGTGGGTCAGACACTTGCTTGTTT	78	103
Pten	Phosphatidylinositol-3,4,5-trisphosphate 3-phosphatase and dual-specificity protein phosphatase PTEN	May be involved in regulation of phosphoinositide 3-kinase and Akt kinase mediated antiapoptotic pathways	NM_031606	ENSRNOG00000020723	qRnoCED0007406	CCACCACAGCTAGAACTTATCAAACCCTTTTGTGAAGATCTTGACCAATGGCTAAGTGAAGACGACAATCATGTTGCAGCAATTCACT	88	97
Rag1	V(D)J recombination-activating protein 1	Expressed in intestinal intraepithelial T lymphocytes	Not Available	ENSRNOG00000004630	qRnoCED0004854	TCTTCCTCCATGTCCATCAAAGCAGACACCAAAGCAGAGTCATAGCGGAACCTCTTTGCAATTGTGTCCACTGGGTATTCATCTACGGAGGAGGC	95	107
Rplp0	60S acidic ribosomal protein P0	Member of the ribosomal protein family; has similarity to other P ribosomal proteins over the carboxyl terminal sequence	NM_022402	ENSRNOG00000001148	qRnoCED0005242	TAAGCAGGCTGACTTGGTGTGAGGGGCTTAGTCGAAGAGACCGAATCCCATGTCCTCATCGGATTCCTCCGACTCTTCCTTTGCTTCGACCTTGGCT	97	100

Rplp2	60S acidic ribosomal protein P2	Component of the ribosome	NM_001030021	ENSRNOG00000002116	qRnoCED0004638	CGCTACGTTGCCTCTTATCTGCTGGCCGCCCTCGGGGGCAACTCCAATCCCAGCGCCAAA	60	105
S100b	Protein S100-B	Binds GTPase activating protein IQGAP1; may play a role in cell membrane rearrangement	NM_013191	ENSRNOG00000001295	qRnoCED0002640	AGACGTCTACTGAGCAGAATTTTGATTCTCGGTCGTGAGTTAGGATGCTAATACTTAGTGTGCGAGATCTAATCGTTCCACCAGTTAGAAC	91	97
Sele	E-selectin	Facilitates recruitment of leukocytes into sites of inflammation; plays a role in cell adhesion	Not Available	ENSRNOG00000002723	qRnoCED0003049	TGATGAAGCAAGTGCGTATTGTCAACGGGACTACACACATCTGGTGGCGATTCAGAACAAGGAAGAGATCAATTACCT	78	101
Slc11a1	Natural resistance-associated macrophage protein 1	Putative metal ion transporter; mouse homolog is a macrophage protein associated with resistance or susceptibility to intracellular pathogens	NM_001031658	ENSRNOG00000014956	qRnoCED0006416	TCTAGAGAGGTAGACAGAACTCGCCGGGGGGATGTTCGAGAAGCCAACATGTACTTCCTGACTGAGGCCACCATCGCCCTCTTCGTGTCATTCATCATCAACCTCTTCGT	110	99
Spp1	Osteopontin	A cytokine that upregulates expression of interferon-gamma and interleukin-12.	NM_012881	ENSRNOG00000043451	qRnoCED0009101	GATTCATCGGAATGGTGAGATTCGTCAGATTCATCCGAGTTCACAGAATCCTCGCTCTCTGCAT	64	99
Stat1	Signal transducer and activator of transcription 1 isoform alpha	Component of the IFN-gamma receptor signaling pathway and other signaling pathways; plays a role in development of cytokine resistance	Not Available	ENSRNOG00000014079	qRnoCID0001659	AGTCATATTCATCTTGTAAGTCTTCTAGGGTCTTGATTTCATGCTCTATGCACATGACTTGGTCCTTCACATTTCTGACTTTACTGTCCAGCTCCTTCT	99	99
Stat3	Signal transducer and activator of transcription 3	Transcription factor that plays a role in induction of gene expression during acute phase response	Not Available	ENSRNOG00000019742	qRnoCID0006149	CTATACTGCTGGTCGATCTCGCCCAAGAGGTTATGAAACACCAGAGTGGCGTGTGACTCTTTGCTGGCTGCATATGCCCAATCTTGGCTCTCAATCCAAG	100	98
Stat4	Signal transducer and activator of transcription 4	This protein is essential for mediating responses to IL12 in lymphocytes, and regulating the differentiation of T helper cells.	Not Available	ENSRNOG00000050942	qRnoCID0001087	TCCAGTTGCTGTCTAAGTTGGAAAAGACTCTCTGCCAGTAGGGTAAAGCAGTTCTGAAGCTGGTCCAACCCGTTGTGGAGCGGGCCACCAATGCAGGCGATCTGTTGCCGCTTCTTC	117	93
Stat6	Signal transducer and transcription activator 6	This protein plays a central role in exerting IL4 mediated biological responses. It is found to induce the expression of BCL2L1/BCL-X(L), which is responsible for the anti-apoptotic activity of IL4.	Not Available	ENSRNOG00000025023	qRnoCED0001198	GACTGCTACCAGAACACTTCCTGTTCCTGGCCCAGAAGATCTTCAATGACAACAGCCTTAGCATAGAGGCCTTTCAGCACCGCTGTGTGTCTTGGTCACAGTTCAAC	107	94
Tbp	TATA-box-binding protein	Mouse homolog binds to TATA box promoter element; involved in activation of eukaryotic	NM_001004198	ENSRNOG00000001489	qRnoCID0057007	TCCTTCACCAATGACTCCTATGACCCCTATCACTCCTGCCACACCAGCCTCTGAGAGCTCTGGGATTGTACCACAGCTCCAAAATATTGTATCCACCGTGAATCTTG	107	95

		genes [RGD, Feb 2006]						
Tbx21	T-box transcription factor TBX21	A Th1 cell-specific transcription factor that controls the expression of the hallmark Th1 cytokine, interferon-gamma (IFNG)	Not Available	ENSRNOG00000009427	qRnoCID0006395	TGGTACTTATGGAGGGACTGCAGGACGATCATCTGGGTCACATTGTTGGAAGCCCCCTTGTTGTTGGTAAGCTTTAGTTTCCCAAATGAAACTTCCTGTCGC	102	97
Tfrc	Transferrin receptor protein 1	Receptor for transferrin; involved in regulating stellate cell activation	NM_022712	ENSRNOG00000001766	qRnoCID0003700	AGCCTCACGAGGAGTATATGTATTCTGGCTCAGCTGCTTGATGATGTCAGTGAACTCTATGGAATT	66	96
Timp1	Metalloproteinase inhibitor 1	Acts as an inhibitor of metalloprotease activity; may play a role in vascular tissue remodeling	NM_053819	ENSRNOG00000010208	qRnoCID0004258	CGTCGAATCCTTTGAGCATCTTAGTCATCTTGATCTCATAACGCTGGTATAAGGTGGTCTCGATGATTTCTGGGGAACCCATGAATTTAGCCCTTATAACCAGGTCCGAGTTGCAGAAA	119	103
Tlr2	Toll-like receptor 2 precursor	A cell-surface protein that can form heterodimers with other TLR family members to recognize conserved molecules derived from microorganisms known as pathogen-associated molecular patterns (PAMPs) and also promotes apoptosis in response to bacterial lipoproteins.	Not Available	ENSRNOG00000009822	qRnoCED0003882	GTCAGGTGATAGATGTCTCTAATTGACTGTAGACTTCGGGACTCATAGTTGCCGAGACTTAATACCTGAATTTCAAGTTCGTTGAGAGAGGTC	93	91
Tlr3	Toll-like receptor 3 precursor	Human homolog induces activation of NF-kappaB; mouse homolog plays a role in the production of inflammatory cytokines	Not Available	ENSRNOG00000021726	qRnoCED0006983	AAGGTGTTCAAGAACTTATTCGAACTCAAGAGCATCAATCTAGGACTGAATAATTTAAACACGCTTCTACCATCCATTTTTGATGACCAGACATCTCTAAG	101	100
Tlr4	Toll-like receptor 4 precursor	Receptor that functions as the major upstream sensor for hemorrhagic shock and lipopolysaccharide	Not Available	ENSRNOG00000010522	qRnoCED0002945	GGTCTAGAAGAGCTGGAATACCTGGACTTTCAGCACTCCACTTTAAAAAAGGTCACAGAATTCTCAGTGTTCTTATCTCTTGAAAAACTTCTTTACCTTGACATCTCTTACACTAAT	117	102
Tlr5	Toll-like receptor 5	Recognizes bacterial flagellin, the principal component of bacterial flagella and a virulence factor. The activation of this receptor mobilizes the nuclear factor NF-kappaB, which in turn activates a host of inflammatory-related target genes.	Not Available	ENSRNOG00000022067	qRnoCED0006599	GAGTTCGGTGCTACAACTGGACCTTTCACACGGCTATATCTTCTCCTTGAACCCCCGACTGTTTGAGACGCTGAAGGATTTGAAGAAGCTGAACCTTGCCTTCAACAAGAT	111	102
Tlr6	Toll-like receptor 6	Interacts with toll-like receptor 2 to mediate cellular response to bacterial lipoproteins.	Not Available	ENSRNOG00000002161	qRnoCED0009346	CGATAACTGAGAGCATAAGCAGGGAGACATTTATTTACGTGGAGACGGTGTTGAAGTCACTGAAGATAGAG	119	97

						CATGTCACAAACCAAGTGTTCCTC TTTGTGAAGGATGCACTATATTCT		
Tlr7	Toll-like receptor 7	TLR7 senses single-stranded RNA oligonucleotides containing guanosine- and uridine-rich sequences from RNA viruses, a recognition occuring in the endosomes of plasmacytoid dendritic cells and B cells.	NM_001097582	ENSRNOG00000004249	qRnoCED0006963	GAATCTGCTCAACTTAGAGGAATT AGATATCTCCAGAAATTCCCTGAA TTCCGTGCCTCCTGGAGTTTTTGA GGGTATGCCACCGAATCTAACGA CTCTCTCC	103	99
Tlr9	Toll-like receptor 9 precursor	TLR9 mediates cellular response to unmethylated CpG dinucleotides in bacterial DNA to mount an innate immune response.	Not Available	ENSRNOG00000048161	qRnoCED0009119	CTCAGCCATAACATCCTCAAGACT GTGGATCGCTCCTGGTTTGGGCC CATTGTGATGAACCTGACGG	67	99
Tnf	Tumor necrosis factor Tumor necrosis factor, membrane form Intracellular domain 1 Intracellular domain 2 C-domain 1 C-domain 2 Tumor necrosis factor, soluble form	Acts as a cytokine; binds TNF receptors; plays a role in regulation of cell proliferation, induction of apoptosis, and inflammatory response	NM_012675	ENSRNOG00000000837	qRnoCED0009117	TGGAGTCATTGCTCTGTGAGGCG ACTGGCGTGTTCATCCGTTCTCTA CCCAGCCCCTGTCCCCGACTCTG ACCCCCATTACTCTGACCCCTTTA TCGTCTACTCCTCAGAGC	112	98
Tp53	Cellular tumor antigen p53	Responds to diverse cellular stresses to regulate target genes that induce cell cycle arrest, apoptosis, senescence, DNA repair, or changes in metabolism.	NM_030989	ENSRNOG00000010756	qRnoCED0004065	ATCCTATCCGGTCAGTTGTTGGAC CTGGCACCTACAGTGAAATTTCAC CCCACCCCACCGCCTGTAAGATT CTATCTTGGGCCCTCATACGATCT GTATCCTCCAGGACC	110	98
Traf6	TNF receptor-associated factor 6	TRAF proteins are associated with, and mediate signal transduction from, members of the TNF receptor superfamily	Not Available	ENSRNOG00000004639	qRnoCED0005912	TGTAAGACTGTGGTCATGTGGTTG CCAACTGTTCAGTGTGACTGTCAT GTAACCTTTCTTGTCTGTTCAGTAT AGCTTGGTTTCCACAGCCTGTCGC ACATCTTCTGTTGCTTGCAA	117	99
Trem1	Triggering receptor expressed on myeloid cells 1	Amplifies neutrophil and monocyte-mediated inflammatory responses triggered by bacterial and fungal infections by stimulating release of proinflammatory chemokines and cytokines	Not Available	ENSRNOG00000022859	qRnoCID0008319	ACAACATTGTATGTGGAGACACTC GTAGGATCTGTCCCATTGTTGATG GTGACTCCAGGATCAGGAGAGGA AACAACAGCAGTGGACTTG	90	104
